# Cost‐Effective Synthesis of Fe_5_C_2_ Catalyst From Nanosized Zero‐Valent Iron to Achieve Efficient Photothermocatalytic CO Hydrogenation to Light Olefins

**DOI:** 10.1002/advs.202410215

**Published:** 2024-12-02

**Authors:** Yuqing Xu, Yuan Li, Ruizhe Li, Hua Xu, Shuxin Ouyang, Hong Yuan

**Affiliations:** ^1^ Engineering Research Center of Photoenergy Utilization for Pollution Control and Carbon Reduction Ministry of Education College of Chemistry Central China Normal University Wuhan 430079 P. R. China; ^2^ School of Chemistry and Environmental Engineering Wuhan Institute of Technology Wuhan 430205 P. R. China; ^3^ Wuhan Institute of Photochemistry and Technology Wuhan 430083 P. R. China

**Keywords:** CO Hydrogenation, Fe_5_C_2_ Catalysts, Light Olefins, Nanosized Zero‐Valent Iron, Photothermocatalysis

## Abstract

Fe‐based catalysts are commonly applied in the process of Fischer−Tropsch synthesis (FTS) to olefins, with Hägg iron carbide (Fe_5_C_2_) recognized as the primary active phase. However, iron carbonyls, the raw materials for wet chemical synthesis of Fe_5_C_2_, are expensive and toxic, which limits large‐scale preparation. Here, a cost‐effective and versatile method is proposed for the synthesis of Fe_5_C_2_ nanoparticles (NPs) with nanosized zero‐valent iron (abbreviated as NZVI, prepared by reducing iron salts or ball‐milling iron powder) instead of iron carbonyls, achieving a cost reduction of 76.8%. Experimental characterizations revealed that NZVI obtained from the reduction of iron salts can catalyze the cracking of octadecylamine to form a carbonized atmosphere, thus realizing the phase transition of Fe into Fe_5_C_2_. The optimized Fe_5_C_2_ catalyst is employed in the photothermocatalytic FTS process, achieving a light olefins selectivity of 54.2% in hydrocarbons, with a CO conversion of 24.3%. Furthermore, it is proved that the particle size and surface oxide state of NZVI can impact the synthesis of Fe_5_C_2_. This study demonstrates a cost‐effective method for the large‐scale preparation of the Fe_5_C_2_ catalyst.

## Graphical abstract

The Fe_5_C_2_ catalyst is synthesized from nanosized zero‐valent iron, through combining a ball‐milling with wet‐chemical carburization approach, which resulted in a 76.8% reduction in costs compared with using Fe_2_(CO)_9_ as iron source. The obtained low‐cost Fe_5_C_2_ catalyst delivered a high light olefins selectivity of 54.2% for CO hydrogenation under light irradiation.

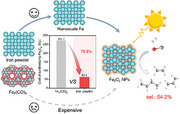

## 1 Introduction

Fischer−Tropsch synthesis (FTS), which converts syngas containing carbon monoxide (CO) and hydrogen (H_2_) into hydrocarbons, is a sustainable pathway for the production of value‐added chemicals.^[^ ^1^ ^]^ Metal‐based catalysts (e.g., Fe, Co, Ni and Ru), are commonly employed in the FTS process for the synthesis of hydrocarbons (like light olefins and liquid fuels).^[^ ^1^, ^2^ ^]^ Among them, many Fe‐based catalysts have exhibited excellent catalytic performance for light olefins (C_2‐4_^=^) during the FTS process; however, Co‐based and Ru‐based catalysts are more inclined to generate paraffins than Fe‐based catalysts.^[^ ^2^, ^3^ ^]^ Meanwhile, Fe‐based catalysts, renowned for their low cost and remarkable selectivity toward light olefins, are extensively employed in the Fischer−Tropsch synthesis to olefins (FTO) process. The reactivity and selectivity of the Fe‐based catalysts in FTS are related to the properties of iron carbides, including χ‐Fe_5_C_2_, θ‐Fe_3_C, ε‐Fe_2_C, ε‐Fe_2.2_C, and Fe_7_C_3_.^[^ ^4^ ^]^ Generally, χ‐Fe_5_C_2_ is widely recognized as the active phase in FTO, in that superior intrinsic catalytic activity and selectivity toward olefins.^[^ ^1^, ^5^ ^]^ However, a large amount of energy input associated with the high temperatures (230−450 °C) and pressures (2–5 MPa) was required to provide thermal energy to drive the Fe‐based FTO process.^[^ ^6^ ^]^ Compared to conventional thermocatalytic strategy, photothermocatalysis utilizes green and sustainable solar energy to initiate chemical reactions through the light‐to‐heat conversion on the catalyst surface, reducing dependence on fossil fuels.^[^ ^7^ ^]^ Recently, Ma and co‐workers reported that oxygen‐decorated Fe_5_C_2_ exhibited an outstanding selectivity for light olefins (55.5%, carbon dioxide (CO_2_) free) with 49.5% CO conversion under light irradiation.^[^ ^8^ ^]^ Later, we found that Fe_5_C_2_/NC600 (a substrate of N‐doped carbon) achieved a selectivity of light olefins (55.3%, CO_2_ free) with a CO conversion of ≈22.3% in the FTO process at a low photothermal temperature of 250 °C.^[^ ^9^ ^]^

The gas‐solid carburization method is commonly employed in the synthesis of metal carbides through reactions between metals and carbon sources.^[^ ^4^, ^5^, ^10^ ^]^ Tang and co‐workers studied the structural evolution of Fe_3_O_4_ under different thermal treatment environments through in situ analyses.^[^ ^11^ ^]^ The results showed that a carbon‐rich phase, especially Fe_2_C, tended to form under high carbon permeation conditions, while under conditions of low carbon permeation, the preference was for the Fe_5_C_2_ formation. In addition to the reaction atmosphere, it was also found that the FeC_x_ phase was related to the reaction temperature. Notably, Fe_2_C exhibited instability above 350 °C, leading to its transformation into Fe_3_C. Currently, the gas‐solid carburization method with the advantages of simple operation is widely applied in the synthesis of iron carbide. However, the preparation of pure‐phase FeC_x_ catalysts for FTS process via the gas‐solid carburization method demands rigorous regulation of various factors, such as the precursor,^[^ ^12^ ^]^ pretreatment atmosphere, temperature,^[^ ^13^ ^]^ and pressure. The wet‐chemical synthesis method offers a facile and practical route to control the composition and morphology of catalysts.^[^ ^4^, ^14^ ^]^ Ma and co‐workers reported a wet chemical method to synthesize Fe_5_C_2_ nanoparticles (NPs), which involved the reaction between iron carbonyls and octadecylamine in the presence of an inducing agent. The results of extended X‐ray absorption fine structure (EXAFS) confirmed that a pure‐phase Fe_5_C_2_ was synthesized successfully.^[^ ^5^ ^]^ Subsequently, many researchers synthesized modified Fe_5_C_2_‐based catalysts using iron carbonyls (such as Fe(CO)_5_ and Fe_2_(CO)_9_) as raw materials.^[^ ^15^ ^]^ Nevertheless, the widespread utilization of iron carbonyls as the raw materials of Fe_5_C_2_ has been hindered by their high cost and toxicity, constraining the industrial‐scale production of Fe_5_C_2_ catalysts. Hence, it is necessary to develop a cost‐effective and environmentally friendly iron source to replace expensive and toxic iron carbonyls to prepare Fe_5_C_2_ catalyst for the FTS reaction.

Herein, we proposed an economical and versatile approach for synthesizing Fe_5_C_2_ from NZVI prepared by reducing iron salts or ball‐milling iron powder. Various characterizations were employed to monitor the transformation of the reaction system during the carbonization process. The results demonstrated that nanosized zero‐valent iron (NZVI) not only acted as the iron source but also catalyzed the decomposition of octadecylamine, resulting in a reduction and carbonization atmosphere. The powder X‐ray diffraction (XRD), X‐ray photoelectron spectroscopy (XPS) and transmission electron microscope (TEM) results demonstrated that Fe_5_C_2_ NPs were successfully synthesized. The obtained Fe_5_C_2_ catalyst was used in the photothermocatalytic FTO process, achieving a selectivity of 54.2% (CO_2_ free) for light olefins, with a CO conversion of 24.3% at 340 °C. When ferrous sulfate heptahydrate (FeSO_4_·7H_2_O) was employed as the iron salts for the synthesis of Fe_5_C_2_, the synthetic cost was reduced by 76.8% compared to that of Fe_2_(CO)_9_ (Tables S1 and S2, Supporting Information). The present work provides a cost‐effective approach for the large‐scale synthesis of Fe_5_C_2_ catalysts.

## 2 Results and Discussion

### 2.1 Morphology and Structure of Fe‐T precursors and Fe_5_C_2_‐T catalysts

In this study, the Fe_5_C_2_ catalysts were synthesized by a two‐step method. Using FeSO_4_·7H_2_O as the typical iron salt, FeSO_4_·7H_2_O was first reduced by sodium borohydride (NaBH_4_) to prepare raw precursors (metallic Fe). XRD patterns of the raw precursors indicated that the diffraction peaks were consistent with those of metallic Fe (Figure S1a, Supporting Information), confirming that the FeSO_4_·7H_2_O had been successfully reduced to Fe. In addition, metallic Fe could be prepared by reducing other iron salts, including ferrous chloride (FeCl_2_), ferric chloride (FeCl_3_), ferric sulfate (Fe_2_(SO_4_)_3_), ferrous acetate ((CH_3_COO)_2_Fe) and ferrous oxalate (FeC_2_O_4_) (Figure S2, Supporting Information). H_2_ temperature‐programmed reduction (H_2_‐TPR) results of the metallic Fe showed two reduction peaks, the low‐temperature reduction peak at 150−285 °C, which indicated the reduction of Fe_2_O_3_ into Fe_3_O_4_ and the high‐temperature reduction peak of 400−800 °C corresponding to the reduction of Fe_3_O_4_‐FeO‐Fe (Figure S3, Supporting Information).^[^ ^16^ ^]^ It suggested the presence of a small amount of iron oxides on the surface of the NZVI, with quantities below the detection limit of XRD technique.

**Figure 1:**
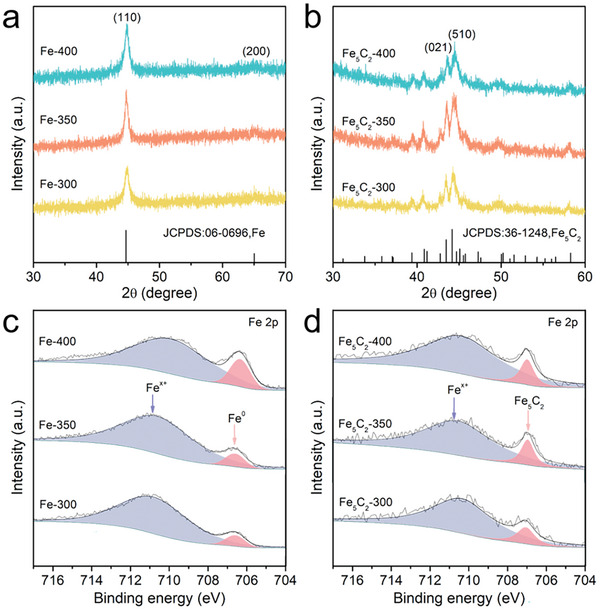
XRD patterns of a) Fe‐T precursors and b) Fe_5_C_2_‐T catalysts. Fe 2p XPS spectra for c) Fe‐T precursors and d) Fe_5_C_2_‐T catalysts.

To obtain a higher reduction state of the metallic Fe, the raw precursor was treated under an H_2_/Ar atmosphere at different temperatures (300, 350 and 400 °C), and the obtained samples were named as Fe‐T (where T represented the reduction temperature, 300, 350 and 400 °C). XRD patterns were used to investigate the phase composition of the Fe‐T samples. As shown in **Figure** **1**a, the XRD patterns of Fe‐T showed that its diffraction peaks at 44.6° and 65.0° were assigned to the (110) and (200) planes of Fe (JCPDS No. 06–0696), respectively. This proved the formation of Fe with good crystallinity. Next, the Fe and Fe‐T precursors were used as the iron sources for wet chemistry synthesis. The octadecylamine was used as the organic solvent and carbon sources, and the solvent was heated to 350 °C under the induction of hexadecyl trimethyl ammonium bromide to prepare the Fe_5_C_2_ catalyst. The obtained catalysts were named as Fe_5_C_2_ and Fe_5_C_2_‐T (T represented the reduction temperature of Fe precursor, 300, 350 and 400 °C), respectively. The XRD patterns showed that the diffraction peaks of Fe_5_C_2_ and Fe_5_C_2_‐T catalysts were consistent with those of χ‐Fe_5_C_2_ (JCPDS No. 36–1248), and the characteristic peaks at 43.5° and 44.4° correspond to the (021) and (510) planes of χ‐Fe_5_C_2_, respectively (Figure 1b; Figure S1b, Supporting Information). This result proved the successful preparation of Fe_5_C_2_ catalyst. The mass percentages of Fe in Fe_5_C_2_‐T catalysts were detected by Inductively coupled plasma‐optical emission spectrometry (ICP‐OES) and the values were summarized in Table S3 (Supporting Information). The chemical state of the iron species in the precursor and catalysts were determined by XPS. As shown in Figure 1c, the peaks observed at a binding energy position of 706.7 eV in the XPS spectra of Fe‐T samples could be attributed to metallic Fe while the peaks of 710.5 eV indicated the presence of iron oxide (Fe^x+^), which was similar to the XPS spectra of Fe (Figure S4c, Supporting Information).^[^ ^17^ ^]^ According to XPS survey spectra, the O content of Fe‐350 was lower than that of Fe without H_2_ reduction (36.4 vs 46.0%, Figure S4 and Table S4, Supporting Information). After the wet chemistry synthesis, the XPS spectra showed that the peaks at 707.1 eV in the Fe 2p spectra of Fe_5_C_2_‐T catalysts could be assigned to Fe_5_C_2_, while the peak at 710.2 eV was associated with iron oxide (Fe^x+^) (Figure 1d).^[^ ^18^ ^]^ These results inferred that amorphous iron oxides were formed on the surface of Fe_5_C_2_‐T during the characterizations.

**Figure 2:**
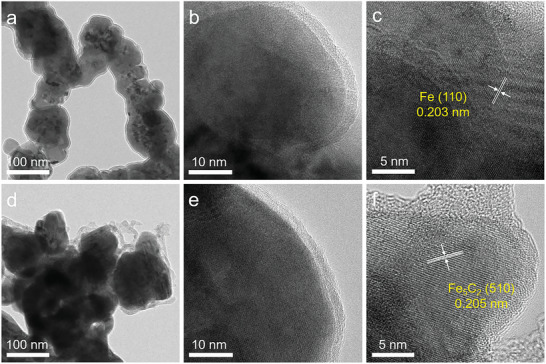
Characterization of Fe‐350 precursor and fresh Fe_5_C_2_‐350 catalyst. a) TEM images, b,c) HRTEM images for the Fe‐350 precursor. d) TEM images, e,f) HRTEM images for the Fe_5_C_2_‐350 catalyst.

The morphology and size of the typical precursor (Fe‐350) and catalyst (Fe_5_C_2_‐350) were further characterized by TEM. As shown in **Figure** **2**a and Figure S5 (Supporting Information), NZVI with a chain‐like structure was observed in the Fe‐350, and the average size of NZVI on the chain‐like structure was 90.2 nm. The surface oxide state of the Fe‐350 was observed in the high‐resolution transmission electron microscope (HRTEM) image (Figure 2b), which agreed with the XPS and H_2_‐TPR analyses (Figure 1c; Figure S3, Supporting Information). The lattice fringe spacing of 0.203 nm could be attributed to the (110) plane of Fe (Figure 2c), consistent with the XRD results (Figure 1a). TEM images of Fe‐300 and Fe‐400 showed that the average size of Fe was 93.8 and 92.7 nm, respectively, which was close to that of Fe‐350 (Figure S6, Supporting Information). Meanwhile, the morphology of Fe‐T displayed a similar chain‐like structure. The HRTEM images of Fe‐300 and Fe‐400 showed (110) lattice fringes of metallic Fe. Furthermore, the morphology of Fe_5_C_2_‐T catalysts synthesized by wet chemistry synthesis was characterized (Figure 2a−d). TEM images of Fe_5_C_2_‐350 revealed a morphological transformation of Fe‐350 after the wet chemical method, offering evidence for the existence of Fe_5_C_2_‐350 nanoparticles. As shown in Figure 2f, the lattice fringe spacing of 0.205 nm could be assigned to the (510) crystal plane of Fe_5_C_2_. The average particle diameter of the Fe_5_C_2_‐T catalysts was ≈109.4 nm (Figure S7, Supporting Information). Meanwhile, the HRTEM images of Fe_5_C_2_‐300 and Fe_5_C_2_‐400 reveal that the Fe_5_C_2_ nanoparticles exhibite similar particle size and morphology to those of the Fe_5_C_2_‐350 catalyst (Figure S8, Supporting Information). Characterizations, including XRD, XPS and TEM confirmed that the NZVI obtained from iron salts could be successfully converted into Fe_5_C_2_ catalyst.

### 2.2 Synthetic Mechanism

**Figure 3:**
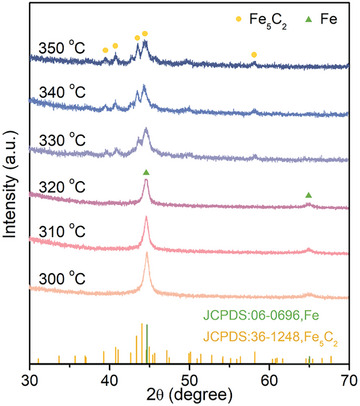
XRD patterns of the crystal phase transformation during the carbonization process from 300 to 350 °C.

The raw precursor (NZVI) served as the iron source of Fe_5_C_2_ in the wet chemical method to explore the synthetic mechanism. To investigate the evolution of the Fe phase, the crystal phase transformation was monitored by XRD during the wet chemical synthesis process. Before the synthetic system was heated, the main Fe phase was metallic Fe (Figure S1, Supporting Information). As the temperature of the synthetic system increase above 300 °C, the intensity of the diffraction peaks corresponding to metallic Fe gradually decreased, and the peaks for metallic Fe completely disappeared when the synthetic system reached 330 °C (**Figure** **3**). Meanwhile, new diffraction peaks appeared, which can be assigned to the Fe_5_C_2_ phase (JCPDS No. 36–1248). With the temperature of synthetic system further raised to 350 °C, the intensity of diffraction peaks stabilized. Therefore, Fe_5_C_2_ was formed in the temperature range of 330−350 °C during the wet chemical synthesis.

**Figure 4:**
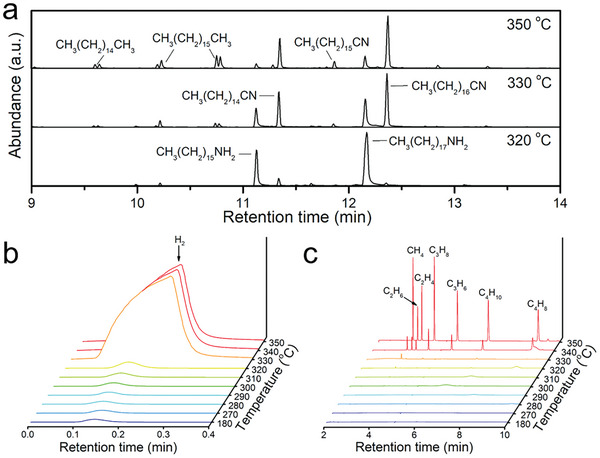
GC‐MS chromatograms obtained from reaction solutions extracted at a) 320, 330, and 350 °C during the wet chemical synthesis process. GC chromatograms of the gas phase in the wet chemical synthesis process. b) H_2_ detected by a TCD detector and c) the hydrocarbons (C_1_‐C_4_) detected by an FID detector.

To identify the carbon sources for the carbonization of Fe precursors, the compositions in the solvent and the gaseous products in the synthetic process were detected at the same time. First, the compositions in solvent were detected by gas chromatography‐mass spectrometry (GC‐MS). When the temperature of the synthetic system was at 180 °C, the GC‐MS chromatograms showed that the compositions in solvent were divided into C_18_H_37_NH_2_ (retention time (t_R_) ≈12.2) and C_16_H_33_NH_2_ (impurity, t_R_ ≈11.1) (Figure S9, Supporting Information). As shown in **Figure** **4**a, even when the temperature reached 320 °C, it was observed that the chemical composition of the solution remained stable. When the temperature was further raised to 330 °C, the peaks due to C_17_H_35_CN (t_R_ ≈12.4) and C_15_H_31_CN (t_R_ ≈11.3) appeared, which demonstrated a transition from −NH_2_ to −CN during the reaction. Subsequently, when the temperature was further increased from 330 to 350 °C, the peak of C_18_H_37_NH_2_ solvent almost disappeared, while the peaks of C_17_H_35_CN and C_15_H_31_CN increased gradually. Additionally, the shorter carbon chain nitriles (C_16_H_33_CN) and hydrocarbons (such as C_17_H_36_ and C_16_H_34_) was detected by the chromatograms. These results indicated that C_18_H_37_NH_2_ was almost completely converted into C_17_H_35_CN, followed by a C─C bond cleavage process of the C_17_H_35_CN. In comparison, when the control synthesis without NZVI was also heated to 350 °C, the chromatogram analysis indicated that only the original solvent C_18_H_37_NH_2_ was detected, without other pyrolysis products (Figure S10, Supporting Information). This observation suggested that the NZVI played a vital role in catalyzing the dehydrogenation of −NH_2_ and cleavage of C─C bonds in the temperature range of 330−350 °C.

Further, the gaseous products in the synthesis process were detected by gas chromatography (GC). The TCD detector of GC was used to detect the H_2_ signal while the FID detector of GC was used to detect hydrocarbons. During the reaction, a signal peak of H_2_ appeared when the synthetic temperature reached 330 °C, and the intensity of the signal peak raised with increasing the temperature (Figure 4b), which was not observed in the control experiment (Figure S11, Supporting Information). These phenomena were consistent with the detection of C_17_H_35_CN in the GC‐MS results at 330 °C, which further confirmed that the NZVI catalyzed the dehydrogenation of C_18_H_37_NH_2_ (C_18_H_37_NH_2_ → C_17_H_35_CN + H_2_). Combined with the results of H_2_‐TPR (Figure S3, Supporting Information), XRD (Figure 1a) and XPS (Figure 1c), it was inferred that the amorphous iron oxides could be reduced by H_2_. The chromatograms from the FID detector displayed signals of small amounts of short‐chain hydrocarbons, including methane (CH_4_), ethane (C_2_H_6_), ethene (C_2_H_4_), propane (C_3_H_8_), propene (C_3_H_6_), butane (C_4_H_10_) and butene (C_4_H_8_), appeared at 330 °C, and the intensity of peak raised with increasing the synthetic temperature (Figure 4c). The above C_1_‐C_4_ hydrocarbons were produced by the C−C bond cleavage of C_17_H_35_CN and other intermediates at temperatures exceeding 330 °C (the corresponding fragments had been detected by GC‐MS). What's more, the NZVI began to be carbonized to form Fe_5_C_2_ when the C_1_‐C_4_ hydrocarbons appeared (Figure 3). Consequently, it was speculated that the carburization of NZVI might be related to the presence of C_1_‐C_4_ hydrocarbons. To further investigate which components of C_1_‐C_4_ hydrocarbons were crucial to the carbonization of NZVI, some control experiments were designed and executed. The raw materials in the wet chemical method were unchanged, when the temperature of the synthetic system was raised to 300 °C (when the solvent was octadecylamine), maintained the temperature and injected an additional gas (such as CH_4_, C_2_H_4_, C_3_H_8_, C_3_H_6_ or N_2_) into the solution for 1 h.^[^ ^19^ ^]^ As shown in Figure S12 (Supporting Information), the XRD patterns showed that the NZVI could be carbonized into Fe_5_C_2_ when the additional gas was C_2_H_4_, C_3_H_8_ or C_3_H_6_, while the carbonization of NZVI could not be achieved when CH_4_ or N_2_ was injected instead. Therefore, the C_2_H_4_, C_3_H_8_ and C_3_H_6_ among the C_1_‐C_4_ hydrocarbons, were identified to be the main carbon sources for converting NZVI into Fe_5_C_2_ NPs. Combined with the results of XRD, GC‐MS and GC, it was verified that the NZVI, reduced from iron salt, could catalyze the pyrolysis of C_17_H_35_CN at 330 °C to produce carbonization atmosphere for Fe_5_C_2_ formation.

**Figure 5:**
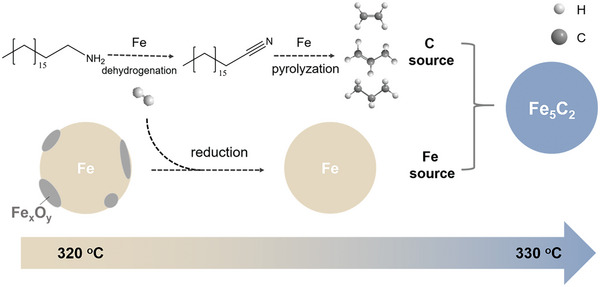
Schematic diagrams of the formation mechanism of Fe_5_C_2_ during the process of carbonization.

According to the above characterization results, we proposed the evolution of Fe species and organic solvent during the wet chemical synthesis. As shown in **Figure** **5**, the interplay between NZVI and the organic solvent played a crucial role in the synthesis of Fe_5_C_2_. The NZVI obtained by the reduction of FeSO_4_·7H_2_O catalyzed the dehydrogenation of C_18_H_37_NH_2_ to C_17_H_35_CN at 320−330 °C. Meanwhile, the H_2_ generated above 330 °C could reduce the surface oxide state of NZVI, according to the H_2_‐TPR results (Figure S3, Supporting Information). With increasing temperature, NZVI could also catalyze the cleavage of C─C bonds in long‐chain nitriles (like C_17_H_35_CN) to produce short‐chain nitriles and C_1_‐C_4_ hydrocarbons at ≈330 °C or higher temperature. The resulting C_2_H_4_, C_3_H_8_ and C_3_H_6_, among the C_1_‐C_4_ hydrocarbons, served as the main carbon sources that further carbonized the NZVI to Fe_5_C_2_ at 330 °C. Therefore, all the above‐mentioned results help us well understand the role of NZVI as an iron source for Fe_5_C_2_ and a driving force for organic solvents to provide reducing agents and carbon sources for Fe_5_C_2_ formation in the wet chemical synthesis.

Based on the understanding of the Fe_5_C_2_ synthetic system, it could be demonstrated that NZVI obtained from the reduction of iron salts with different oxidation states and anionic coordination could act as the iron source to produce Fe_5_C_2_. However, whether the surface oxide state of NZVI could be completely reduced by the obtained H_2_ generated during the wet chemical synthesis process remained uncertain. It was suggested that the unreduced iron oxides remaining on the surface of NZVI might negatively impact the wet chemical synthesis process owing to the unsatisfactory carbonizing ability of iron oxides (Fe^2+^/Fe^3+^),^[^ ^20^ ^]^ which could lead to different surface active phase.

In addition, it was found that the particle size of NZVI could affect the wet chemical synthesis process. The NZVI precursors with different average particle sizes were prepared by a ball‐milling method, and the obtained samples were named as Mx‐Fe (where x represented ball‐milling time, 0−72 h). TEM images showed that the obtained NZVI precursors had an average particle size range of 9−107 nm (Figures S13 and S14, Supporting Information). During the wet chemical synthesis with Mx‐Fe, the H_2_ signal was detected by the TCD detector of GC. As shown in Figure S15 (Supporting Information), the signal peaks of H_2_ appeared above 320 °C, except that of M0‐Fe. Therefore, the phase transition from NZVI to Fe_5_C_2_ can be realized when the average particle sizes of Fe was below 107 nm (Figure S16, Supporting Information). It was worth noting that the Mx‐Fe contained similar oxygen content (39.5 vs 39.1%, Table S5, Supporting Information), which indicated that the difference in the wet chemical synthesis was not ascribed to the surface oxide state of Mx‐Fe. The results indicated that NZVI with an average particle size ranging from 9 to 43 nm could effectively catalyze the dehydrogenation of C_18_H_37_NH_2_.

### 2.3 Catalytic Properties

In the wet chemistry synthesis of Fe_5_C_2_ with NZVI instead of iron carbonyls as the precursor, it facilitated exploring the key factors (such as the NZVI particle size and the surface oxidation state) of the synthesis and its influence on the catalytic performance. According to the above characterizations, M0‐Fe with a large particle size cannot realize the phase transition from NZVI to Fe_5_C_2_ nanoparticles. While using Mx‐Fe precursors (10 ≤ x ≤ 72), Mx‐Fe_5_C_2_ catalysts with different average particle sizes could be successfully synthesized through the wet chemistry method, and the lattice spacing of 0.205 nm was assigned to the Fe_5_C_2_ (510) planes (Figures S17 and S18, Supporting Information). Since the photothermocatalysis could significantly reduce the energy consumption compared with traditional thermocatalysis, a series of photothermocatalytic experiments (the reaction system is exhibited in Figure S19, Supporting Information) was adopted to investigate whether the particle size of obtained Mx‐Fe_5_C_2_ catalysts affected the FTO performance. The catalytic performance of Mx‐Fe_5_C_2_ catalysts was summarized (**Table** **1**, entries 1−5 and Figure S20, Supporting Information), and a volcano‐like evolution of the catalytic activity was observed with increasing ball‐milling time of the precursors. It was observed that M10‐Fe_5_C_2_ exhibited some activity for FTO, with a CO conversion of 23.3%. The highest CO conversion (34.1%) and the excellent selectivity of light olefins (51.7%) were achieved over the M12‐Fe_5_C_2_ catalyst. In a further step, extended ball‐milling times led to a decreased catalytic activity, with CO conversion declining from 34.1% for M12‐Fe_5_C_2_ to 11.5% for M72‐Fe_5_C_2_, while the selectivity of light olefins remained almost constant (50.1−51.7%). The long‐term stability of the M12‐Fe_5_C_2_ catalyst was evaluated in a flow system, demonstrating excellent stability (Figures S21 and S22, Supporting Information). To investigate the mechanism of high CO conversion, the adsorption capacity of CO was characterized by CO temperature‐programmed desorption experiments, which were related to the active sites. As shown in Figure S23 (Supporting Information), the desorption peaks located at 400−450 and 520−570 °C were both ascribed to CO desorption from Fe_5_C_2_, while the desorption peaks above 600 °C corresponded to the reverse Boudouard reaction.^[^ ^9^, ^20^, ^21^ ^]^ Obviously, the desorption peak (569 °C) and the amount of CO (0.72 mmol g^−1^) of M12‐Fe_5_C_2_ were significantly higher than those of other Mx‐Fe_5_C_2_ catalysts, implying that appropriately reducing the particle size of Fe_5_C_2_ enhanced the adsorption ability of active sites. However, with the further increase of ball‐milling time, the desorption peak and the amount of CO of Mx‐Fe_5_C_2_ gradually shifted to lower temperatures. Combined with TEM images (Figure S17, Supporting Information), it can be inferred that excessive ball‐milling time may reduce the dispersion of the catalysts, resulting in a decrease in the adsorption capacity of active sites. Consequently, the Fe_5_C_2_ synthesized from NZVI with an average particle size of ≈19 nm exhibited a strong adsorption capacity for CO, resulting in the optimal catalytic performance.

**Table 1:** Catalytic performance of Fe_5_C_2_ catalysts synthesized from NZVI with different ball‐milling times or iron salts.

Entry^a)^	Catalyst	CO conv. [%]	CO_2_ sel.[%]	Hydrocarbon sel. [%, CO_2_ free]			
				CH_4_	C_2‐4_^=^	C_2‐4_^0^	C_5+_
1	M10‐Fe_5_C_2_	23.3	2.1	28.3	50.7	2.4	18.6
2	M12‐Fe_5_C_2_	34.1	4.1	33.1	51.7	2.7	14.1
3	M24‐Fe_5_C_2_	25.9	2.9	28.1	50.6	3.8	17.5
4	M48‐Fe_5_C_2_	20.6	4.0	26.8	50.1	3.2	19.9
5	M72‐Fe_5_C_2_	11.5	2.5	24.1	50.7	1.6	23.6
6	FeCl_2_‐350	29.8	3.4	31.9	50.0	2.1	16.0
7	FeCl_3_‐350	28.5	6.1	34.0	51.8	3.9	10.3
8	FeSO_4_‐350	24.3	5.1	28.7	54.2	3.2	13.9
9	Fe_2_(SO_4_)_3_‐350	24.3	3.1	33.8	52.1	3.1	11.0
10	(CH_3_COO)_2_Fe‐350	1.7	8.0	33.0	52.6	1.8	12.6
11	FeC_2_O_4_‐350	17.2	4.0	27.8	54.0	3.0	15.2

^a)^ Reaction conditions: reaction atmosphere, CO/H_2_/N_2_ = 20/60/20, 0.18 MPa; catalyst mass, 50 mg; irradiation time, 0.5 h; light source, 300 W Xe lamp (λ = 200−1200 nm, 340 °C).

**Figure 6:**
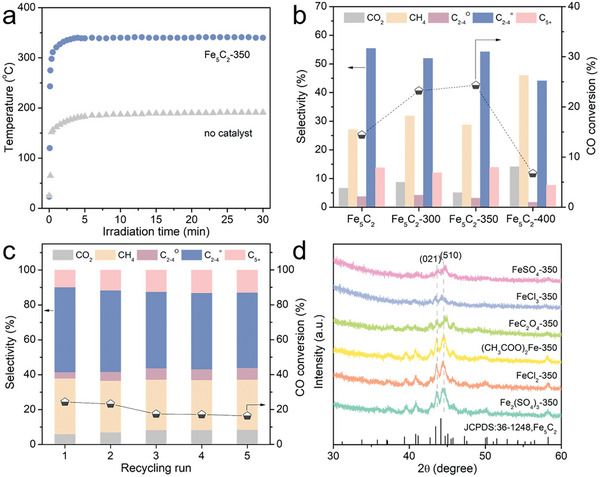
a) Temperature profiles of the Fe_5_C_2_‐350 and no catalyst under the 6.2 W cm^−2^ illumination. b) FTS catalytic performance tests of Fe_5_C_2_‐T catalysts. c) Stability test of Fe_5_C_2_‐350 catalyst for five cycles. d) XRD patterns of Fe_5_C_2_ prepared from different iron salts. All the performance tests were conducted in a closed system: reaction atmosphere, CO/H_2_/N_2_ = 20/60/20, 0.18 MPa; catalyst mass, 50 mg; irradiation time, 0.5 h; light source, 300 W Xe lamp (λ = 200−1200 nm, 340 °C).

From the analysis of the Fe_5_C_2_ synthesis mechanism, it could be seen that in addition to the size effect, the surface oxide state of NZVI also affected the Fe_5_C_2_ synthesis and subsequent catalytic performance. However, the oxygen concentration in Mx‐Fe was nearly identical across the ball milling method (39.5% vs 39.1%, Table S5, Supporting Information). To further investigate the impact of surface oxide state on the photothermocatalytic performance of NZVI, NZVI was prepared via a two‐step reduction process (the reduction of iron salts followed by hydrogen reduction at various temperatures) to obtain the Fe_5_C_2_‐T catalysts with different surface oxide state. Subsequently, the photothermocatalytic properties of the Fe_5_C_2_‐T catalysts were evaluated. As the light absorption characteristics played a crucial role in the performance of photothermal catalysts, ultraviolet‐visible near‐infrared (UV−Vis−NIR) absorption spectra were acquired to assess the light absorption capacity of the catalysts. The UV−Vis−NIR absorption spectra revealed that the as‐prepared Fe_5_C_2_ and Fe_5_C_2_‐T catalysts exhibited excellent light absorption which covered the ultraviolet, visible and near‐infrared light regions (Figure S24, Supporting Information), rendering them ideal materials for photothermocatalysis. In the subsequent experiments, the Fe_5_C_2_‐350 catalyst was taken as a typical example. To manifest the efficiency of light‐to‐heat conversion over Fe_5_C_2_‐350 catalyst in the photothermocatalytic process, the surface temperature of the catalyst was recorded by a directly contacting thermocouple under the illumination of a 300 W Xe lamp with an intensity of 6.2 W cm^−2^. As shown in **Figure** **6**a, under the light irradiation, the surface temperature of Fe_5_C_2_‐350 catalyst increased rapidly within 2 min and gradually stabilized at ≈340 °C after ≈4 min. In the absence of the catalyst, the temperature measured by the thermocouple remained stable at 190 °C. It suggested that the Fe_5_C_2_‐350 catalyst exhibited great efficiency of light‐to‐heat conversion.

The catalytic performances of Fe_5_C_2_‐T and reference catalysts in the FTO reaction were investigated. As shown in Table S6 (Supporting Information) (entry 1), no products were detected in the blank experiment conducted without a catalyst, indicating that a catalyst was crucial for CO hydrogenation. Meanwhile, the precursor (like Fe‐350) was also employed in the photothermocatalytic FTS reaction (Table S6, Supporting Information, entry 2), and exhibited low CO conversion (5.1%) and poor selectivity of light olefins (15.3%). In comparison, pristine Fe_5_C_2_ showed some activity for CO conversion (14.4%), with an excellent selectivity for light olefins of ≈55.4% (Figure 6b). The results indicated that Fe_5_C_2_ was the active phase for the FTO process. In a further step, a volcano‐like evolution of the catalytic activity with increasing reduction temperature of the precursors was observed (Figure 6b). Compared to pristine Fe_5_C_2_ catalysts, both Fe_5_C_2_‐300 and Fe_5_C_2_‐350 catalysts exhibited enhanced catalytic activity, while the selectivity of light olefins remained at a similar level (51.9−54.2%). The highest CO conversion (24.3%) and excellent selectivity of light olefins (54.2%) could be achieved with the Fe_5_C_2_‐350 catalyst. However, when the reduction temperature of the precursors was further increased to 400 °C, both the catalytic activity and selectivity of light olefins suddenly dropped to 6.7% and 44.1%, respectively.

Therefore, the reduction temperature of precursors significantly affected the CO hydrogenation activity of the series of Fe_5_C_2_‐T catalysts. The catalytic performance of the Fe_5_C_2_‐350 catalyst under the different ratios of CO/H_2_ was also tested (Figure S25, Supporting Information). The CO conversion of the Fe_5_C_2_‐350 catalyst rose from 5.3% to 24.3% under reaction conditions of CO/H_2_/N_2_ = 20/60/20, while the selectivity of light olefins remained almost constant (54.2−56.0%) (CO_2_ free). Therefore, the ratio of CO/H_2_/N_2_ = 20/60/20 was chosen as the optimized reaction condition. Moreover, the product distributions of the Fe_5_C_2_ and Fe_5_C_2_‐T catalysts conformed the classical Anderson−Schulz−Flory (ASF) model (Figures S26 and S27, Supporting Information). The stability of the Fe_5_C_2_‐350 catalyst was evaluated by cycling experiments under light irradiation with an intensity of 6.2 W cm^−2^. It demonstrated consistent stability in both CO conversions and light olefins selectivity over five cycles (Figure 6c). Moreover, the XRD pattern of the spent Fe_5_C_2_‐350 catalyst exhibited no obvious changes compared to that before the reaction (Figure S28, Supporting Information), which demonstrated that the catalyst maintained stable catalytic performance in the photothermocatalytic FTO process.

To explore the reasons for the differences in catalytic performance, the composition and structure of the precursors and catalysts were analyzed. The content of Fe in Fe_5_C_2_‐T was almost the same (Table S3, Supporting Information). The HRTEM images of Fe_5_C_2_‐T precluded the effect of particles size on catalytic performance (Figure 2; Figure S7, Supporting Information). According to the XPS results, it was worth noting that the O content of the NZVI was higher than that of nanoscale Fe‐350 before carbonization (Figure S4 and Table S4, Supporting Information), so the higher CO conversion of the Fe_5_C_2_‐350 catalyst than that of other Fe_5_C_2_ catalysts might be explained by the different oxidation states of the NZVI. In addition, the excessive reduction temperature might reduce the surface oxide state of Fe‐T precursor, thus suppressing the catalytic activity of Fe_5_C_2_‐T. Consequently, the Brunauer–Emmett–Teller (BET) surface areas of Fe‐T were tested. As shown in Figure S29 and Table S7 (Supporting Information), the results disclosed that the BET surface areas of Fe‐300 and Fe‐350 were almost the same (7.5−8.1 m^2^ g^−1^) and larger than that of Fe‐400 (5.9 m^2^ g^−1^). It could be signified that the positive effect of reducing the surface oxide state of NZVI in promoting the formation of active phase Fe_5_C_2_ combined with the negative influence of excessive reduction temperature causing the sintering of NZVI led to the volcano‐like evolution of the FTO activity in this case. Hence, it was crucial for selecting a moderate reduction temperature (350 °C) to reduce the precursor. Moreover, it was found that the catalytic performance was also affected by the oxidation states of Fe_5_C_2_ (Figures S30–S32, Supporting Information).

Considering that the wide range of sources of iron salts facilitated the efficient synthesis of Fe_5_C_2_, the universality of different iron salts in the synthesis method was further explored. The Fe_5_C_2_ catalysts were prepared by the same process except that FeSO_4_·7H_2_O was replaced with other iron salts (see the Experimental Section of Supporting Information for details). The iron salts were explored in terms of valence state of iron (such as FeCl_2_ and FeCl_3_, FeSO_4_ and Fe_2_(SO_4_)_3_) and coordination anion of iron (such as FeCl_2_, FeSO_4_, (CH_3_COO)_2_Fe and FeC_2_O_4_). Notably, the XRD patterns of the obtained catalysts were presented in Figure 6d, where the characteristic peaks at 43.5° and 44.4° were assigned as the (021) and (510) planes of Fe_5_C_2_, indicating the successful preparation of the Fe_5_C_2_ catalyst. It demonstrated that iron salts with different valence states or anionic coordination could be reduced to NZVI, and then to be converted into Fe_5_C_2_ by wet chemical synthesis. Under illumination, the catalytic performance of the obtained Fe_5_C_2_ catalysts in the FTO reaction was evaluated and listed in Table 1 (entries 6−11). All of the obtained Fe_5_C_2_ catalysts showed high selectivity for light olefins (50.0−54.2%), while the CO conversions were different when the coordination anion in iron salt was changed. Based on these results, it was proved that NZVI obtained by the reduction of iron salts with different oxidation states and anionic coordination could be used as iron source for Fe_5_C_2_. It indicated that the present synthesis method broadened the selection of raw materials for the synthesis of Fe_5_C_2_ catalyst.

Since reducing the cost of raw materials could promote the industrial production of catalysts, the price of each material for synthesizing Fe_5_C_2_ and the total cost of synthesizing Fe_5_C_2_ were calculated, respectively (summarized in Tables S1 and S2, Supporting Information). The results showed that the cost of Fe_2_(CO)_9_ was the key factor driving up the total synthesis cost of Fe_5_C_2_, making it advantageous to replace Fe_2_(CO)_9_ with low‐cost iron powder or iron salts. Compared to using Fe_2_(CO)_9_ as the iron source, the synthesis cost could be reduced by 76.8% and 75.5% when using iron powder and iron salts, respectively (Table S8 and Figures S33 and S34, Supporting Information). Notably, preparing the NZVI precursor from iron powder using the ball‐milling method avoided the need for environmentally harmful reagents and was straightforward to operate, showing great potential for large‐scale production. Furthermore, the Fe_5_C_2_ catalyst prepared via ball milling and sodium borohydride reduction exhibited the same catalytic performance as Fe_5_C_2_ synthesized from carbonyl iron. In summary, this study provides an economically efficient method for promoting the large‐scale preparation of Fe_5_C_2_ catalysts.

## 3 Conclusion

In summary, an economical and universal strategy for synthesizing Fe_5_C_2_ with NZVI instead of iron carbonyls was developed, achieving a cost reduction of 76.8%. The formation mechanism of Fe_5_C_2_ was investigated by the experimental characterizations including XRD, XPS, H_2_‐TPR, GC and GC‐MS, revealing that NZVI played a vital role in the synthesis of Fe_5_C_2_. Furthermore, Fe_5_C_2_ was identified as the active phase in FTS reaction, with the optimized Fe_5_C_2_‐350 catalyst demonstrating efficient photothermocatalytic activity (CO conversion, 24.3%) and exhibiting a prominent selectivity of light olefins (54.2%, CO_2_ free). The excellent catalytic activity could be attributed to the reduction of the iron oxide on the surface of NZVI during hydrogen reduction, which promoted the carbonization process. In addition, this work demonstrates that the particle sizes of NZVI can affect the synthesis of Fe_5_C_2_ and the catalytic performance of the obtained Fe_5_C_2_. Overall, this study not only presents a cost‐effective, facile and general method for synthesizing Fe_5_C_2_, but also contributes to a better understanding of the wet chemical synthesis process. This strategy is expected to enable the large‐scale production of FTO catalysts for solar energy conversion.

## Conflict of Interest

The authors declare no conflict of interest.

## Supporting information



Supporting Information

## Data Availability

The data that support the findings of this study are available from the corresponding author upon reasonable request.
