# Supplementary material for: Cost‐Effective Synthesis of Fe5C2 Catalyst From Nanosized Zero‐Valent Iron to Achieve Efficient Photothermocatalytic CO Hydrogenation to Light Olefins
Source: Adv Sci (Weinh). 2024 Dec 2;12(4):2410215. doi: 10.1002/advs.202410215 (PMC11775531; doi:10.1002/advs.202410215)
Supplement: Supplementary file 1 — Supporting Information [file ADVS-12-2410215-s001.pdf]

ADVANCED SCIENCE
Open Access

Supporting Information

for *Adv. Sci.*, DOI 10.1002/advs.202410215

Cost-Effective Synthesis of Fe_5C_2 Catalyst From Nanosized Zero-Valent Iron to Achieve Efficient Photothermocatalytic CO Hydrogenation to Light Olefins

Yuqing Xu, Yuan Li, Ruizhe Li, Hua Xu, Shuxin Ouyang* and Hong Yuan*

***Supporting Information* for**

Cost-Effective Synthesis of Fe_5C_2 Catalyst from Nanosized Zero-Valent Iron to Achieve Efficient Photothermocatalytic CO Hydrogenation to Light Olefins

Yuqing Xu,[a]+ Yuan Li,[a]+ Ruizhe Li,[a] Hua Xu,*[b] Shuxin Ouyang*[a][c] and Hong Yuan[a]

[a] Engineering Research Center of Photoenergy Utilization for Pollution Control and Carbon Reduction, Ministry of Education, college of Chemistry, Central China Normal University, Wuhan 430079 (P. R. China).

[b] School of Chemistry and Environmental Engineering, Wuhan Institute of Technology, Wuhan 430205 (P. R. China).

[c] Wuhan Institute of Photochemistry and Technology, Wuhan 430083 (P. R. China)

Corresponding Authors

*Email: oysx@mail.ccnu.edu.cn

XU.Hua@wit.edu.cn

[+] These authors contributed equally to this word.

Table of contents

Experimental Section 5
Materials 5
Synthesis of nanosized Fe-T 5
Synthesis of nanosized Mx-Fe 5
Synthesis of Fe_5C_2-T or Mx-Fe_5C_2 NPs 6
Sample characterizations 6
Photothermocatalytic evaluation 7
Supplementary Figures 8
Figure S1. XRD patterns of Fe and pristine Fe_5C_2. 8
Figure S2. XRD patterns of Fe prepared from different iron salts. 9
Figure S3. H_2-TPR patterns of NZVI. 10
Figure S4. (a) XPS survey spectra and (b) O 1s XPS spectra for Fe and Fe-350 precursors. Fe 2p XPS spectra for precursors (c) Fe and (d) Fe-350. 11
Figure S5. TEM images of Fe-300 and Fe-400. (a) TEM images, (b) and (c) HRTEM images for Fe-300. (d) TEM images, (e) and (f) HRTEM images for Fe-400. 12
Figure S6. Particle size distribution histograms of Fe-T. 13
Figure S7. Particle size distribution histograms of Fe_5C_2-T. 14
Figure S8. TEM images of Fe_5C_2-300 and Fe_5C_2-400 catalysts. (a) TEM images, (b) and (c) HRTEM images for Fe_5C_2-300 catalyst. (d) TEM images, (e) and (f) HRTEM images for Fe_5C_2-400 catalyst. 15
Figure S9. GC-MS chromatograms obtained from the reaction solutions extracted at 180 °C and 310 °C. 16
Figure S10. GC-MS chromatograms obtained from the reaction solutions of the blank experiment at 350 °C without NZVI. 17
Figure S11. GC chromatograms obtained from the wet synthesis process without NZVI detected by the TCD detector. 18
Figure S12. XRD patterns of products generated from control experiments. 19
Figure S13. Characterization of Mx-Fe precursors. TEM images of (a) M0-Fe, (b) M10-Fe, (c) M12-Fe and (d) M72-Fe. HRTEM images of (e) M0-Fe, (f) M10-Fe, (g) M12-Fe and (h) M72-Fe. 20
Figure S14. Particle size distribution histograms of Mx-Fe. 21
Figure S15. GC chromatograms of the H_2 signal in the wet chemical synthesis process of Mx-Fe. 22

Figure S16. XRD patterns of Mx-Fe_5C_2. 23
Figure S17. Characterization of Mx-Fe_5C_2 catalysts. TEM images of (a) M10-Fe_5C_2, (b) M12-Fe_5C_2 and (c) M72-Fe_5C_2. HRTEM images of (d) M10-Fe_5C_2, (e) M12-Fe_5C_2 and (f) M72-Fe_5C_2. 24
Figure S18. Particle size distribution histograms of Mx-Fe_5C_2. 25
Figure S19. Photographs of (a) photothermal reactor and (b) catalytic evaluation system. 26
Figure S20. FTS catalytic performance tests of Mx-Fe_5C_2 catalysts. 27
Figure S21. Stability test of M12-Fe_5C_2 catalyst in a flow reaction system for 10 h. 28
Figure S22. XRD patterns for M12-Fe_5C_2 before the reaction and after 10 h.... 29
Figure S23. CO temperature-programmed desorption profiles of Mx-Fe_5C_2 catalysts. 30
Figure S24. Ultraviolet-visible near-infrared absorption spectra of the Fe_5C_2 and Fe_5C_2-T catalysts.. 31
Figure S25. The photothermocatalytic performance of Fe_5C_2-350 catalyst under different ratio of CO/H_2. 32
Figure S26. Chromatogram of the hydrocarbon products obtained by CO hydrogenation over the Fe_5C_2-350 catalyst for 0.5 h under light irradiation from a 300 W Xe lamp.. 33
Figure S27. The hydrocarbon product distribution obtained over the catalysts under the irradiation of the 300 W Xe lamp: (a) Fe_5C_2, (b) Fe_5C_2-300, (c) Fe_5C_2-350, (d) Fe_5C_2-400. 34
Figure S28. XRD patterns for Fe_5C_2-350 before the reaction and after 5 times. 35
Figure S29. N_2 adsorption-desorption curves of Fe-T samples. 36
Figure S30. XRD patterns of Fe_5C_2 with different surface oxide states. 37
Figure S31. Fe 2p XPS spectra of Fe_5C_2 with different surface oxide states..... 38
Figure S32. Catalytic performances of Fe_5C_2 catalysts with different surface oxide states. 39
Figure S33. Stability test of Fe_5C_2 prepared from $Fe_2(CO)_9$ in a flow reaction system for 10 h.. 40
Figure S34. XRD patterns of Fe_5C_2 prepared from $Fe_2(CO)_9$ after the reaction for a duration of 10 h. 41
Supplementary Tables 42
Table S1. The price of each material for synthesizing Fe_5C_2. 42

Table S2. Comparison of the experimental cost between $Fe_2(CO)_9$, iron powder and iron salts... 43
Table S3. Element contents of Fe_5C_2-T catalysts were detected by ICP. 44
Table S4. Element contents of the Fe and Fe-350 samples............................. 45
Table S5. Element contents of the M12-Fe and M72-Fe samples.................... 46
Table S6. Catalytic performance of blank experiment and Fe-350 sample........ 47
Table S7. Surface area and pore volume dates for Fe-T samples. 48
Table S8. Catalytic performance of Fe_5C_2 obtained from $Fe_2(CO)_9$ or iron powder. 49

Experimental Section

Materials

All reagents were used as received without further processing. Iron (II) sulfate heptahydrate ($FeSO_4 \cdot 7H_2O$, AR), hexane (C_6H_{14}, AR), ethanol (C_2H_5OH, AR) and ethylene glycol ($(CH_2OH)_2$, AR) were purchased from Sinopharm Chemical Reagent Co., Ltd. Sodium borohydride ($NaBH_4$, 98.0%), iron (II) acetate ($(CH_3COOH)_2Fe$, 90.0%) and reduced iron powder (Fe, CP) were purchased from Shanghai Aladdin Biochemical Technology Co., Ltd. Octadecylamine (ODA, 98.0%) was obtained from Beijing HWRK Chemical Co., Ltd. Hexadecyl trimethyl ammonium bromide (CTAB, 99.0%) and iron chloride hexahydrate ($FeCl_3 \cdot 6H_2O$, 99.0%) were obtained from Macklin. Iron sulfate ($Fe_2(SO_4)_3$, AR) was supplied by Meryer. Iron (II) chloride ($FeCl_2$, 99.5%) and ferrous oxalate (FeC_2O_4, 99%) were supplied by Energy Chemical.

Synthesis of nanosized Fe-T

The raw nanosized Fe used as a precursor was prepared by reducing iron salt with sodium borohydride, [1] then further reduced under a H_2/Ar atmosphere at different temperatures. As a typical run, 1.1 g of $FeSO_4 \cdot 7H_2O$ was dissolved in 20 mL of ultrapure water. At room temperature, the excess sodium borohydride solution (0.01 $mol \cdot L^{-1}$) was slowly added into the aqueous $FeSO_4$ solution under stirring (200 rpm). A black precipitate and amounts of bubbles were observed, immediately. After stirring for 5 min, the product was collected by centrifugation (11000 rpm), and washed with ultrapure water four times. The obtained sample was dried overnight in a vacuum freeze-drier. Finally, the nanosized Fe was obtained by reducing the dried sample at different temperatures (300−400 ^{o}C) for 2 h with a ramp rate of 10 ^{o}C per min in a flow of hydrogen (H_2/Ar = 10/90) and named Fe-T (where T represented the reduction temperature). In a further step, the nanosized Fe was prepared by a similar procedure except that $FeSO_4 \cdot 7H_2O$ was replaced with other iron sources (such as $FeCl_3 \cdot 6H_2O$, $Fe_2(SO_4)_3$, $FeCl_2$, $FeSO_4$, $(CH_3COOH)_2Fe$, FeC_2O_4).

Synthesis of nanosized Mx-Fe

The nanosized Fe with different average sizes as precursors were prepared by a ball-milling method. 5 g of reduced iron powder, 20 mL of $(CH_2OH)_2$ and 2 mm diameter

stainless steel balls were put in a planetary ball mill machine in 100 mL stainless steel jars (the Fe-to-balls mass ratio is 1:20). The machine was operated for 0−72 h at 500 rpm, changing the direction of rotation every 1 h. After milling, the samples were washed three times with ethanol. The obtained precursors were freeze-dried overnight under vacuum and named Mx-Fe (where x represented the running time of the ball mill machine).

Synthesis of Fe_5C_2-T or Mx-Fe_5C_2 NPs

The Fe_5C_2 catalysts were synthesized using a wet chemistry method. [2] Typically, 0.113 g of CTAB, 0.288 g of Fe-T or Mx-Fe, and 14.5 g of ODA were mixed in a 100 mL three-neck flask with magnetic stirring under a nitrogen atmosphere. First, the reactants were heated to 180 ^{o}C with a rate of 10 $^{o}C\cdot min^{-1}$ and kept for 10 min, then increased to 350 ^{o}C and held for 10 min. After cooling to 70 ^{o}C, the dark precipitate in the solution was collected by centrifugation (11000 rpm), washed six times with hexane and ethanol, alternately. The obtained catalyst was freeze-dried overnight under vacuum, for further characterization and performance testing. According to the precursor, the corresponding catalysts were named Fe_5C_2-T (T represented the reduction temperature) or Mx-Fe_5C_2 (x represented the running time of the ball mill machine).

Sample characterizations

Powder X-ray diffraction (XRD) patterns were collected on a Bruker D8-Phaser X-ray diffractometer with a Cu Kα radiation source (λ = 0.15418 nm). X-ray photoelectron spectroscopy (XPS) experiments were carried out on a Thermo Scientific Escalab Xi+ system using a monochromatized Al Kα anode. H_2 temperature-programmed reduction (H_2-TPR) was characterized on a Quantachrome ChemBET Pulsar instrument. Inductively coupled plasma-optical emission spectrometry (ICP-OES, Agilent 730) was used to measure the content of various elements in Fe_5C_2-T catalysts. The morphologies of the samples were performed on a transmission electron microscope (TEM, JEOL JEM-2100F, Japan). The light absorption capacity of catalysts was collected from an ultraviolet-visible near-infrared (UV-Vis-NIR) spectroscopy system from 200 to 2500 nm (UV-3600 plus, Shimadzu, Japan). The synthesis mechanism was studied by gas chromatography (GC, Agilent 7890B) and gas chromatography-mass spectrometry (GC-MS, Shimadzu GCMS-QP2020). The Brunauer-Emmett-Teller (BET) surface areas were determined by Quantachome Autosorb-iQ instrument with nitrogen adsorption at 77 K. CO temperature-programmed desorption (CO-TPD) experiments were carried out on a Quantachrome

ChemBET Pulsar instrument.

Photothermocatalytic evaluation

The photothermocatalytic FTO reactions were conducted in a 50 cm^3 stainless steel batch-type reactor chamber with a quartz window for light (Beijing Perfectlight Technology Co., Ltd., PLR-GPTR50). Regarding each reaction, 50 mg of the samples was spread flat around the thermocouple at the center of the reactor. Before the photothermocatalytic evaluation, the reaction system was degassed and purged with syngas ($CO/H_2/N_2$ = 20/60/20) three times, then the syngas was charged into the reaction system until the internal pressure reached 0.18 MPa. The photothermal reaction was initiated by a Xe lamp (Beijing Perfectlight Technology Co., Ltd., PLS-SXE-300DUV, 6.24 W·cm^{-2}, 200−1200 nm). In the course of photothermal reaction, the surface temperature of the catalyst was rapidly heated to 340 ^{o}C within 2 min and then maintained at 340 ^{o}C for 30 min by adjusting the output current of the Xe lamp. After finishing the irradiation, the amounts of hydrocarbons were detected on a gas chromatograph installed with the flame ionization detector (FID) installed (GC, Agilent 7890B). The amounts of CO_2, N_2, O_2, CO and CH_4 were analyzed on the GC installed with the thermal conductivity detector (TCD), as described in our previous work. [3]

The CO conversion rate (C_{CO}), regarded as characteristic of the photothermal catalytic activity, was defined as

$$C_{CO} = \left(1 - \frac{area_{CO}}{area_{N_2}} \times \frac{1}{f_{CO-N_2}}\right) \times 100\%$$

and the selectivity of CH_4 (S_{CH_4}), CO_2 (S_{CO_2}) and C_xH_y ($S_{C_xH_y}$, CO_2 free) were defined as

$$S_{CH_4} = \frac{1}{f_{CH_4-CO_2} \times \frac{area_{CO_2-TCD}}{area_{CH_4-TCD}} + \frac{\sum area_{C_xH_y-FID}}{area_{CH_4-FID}}} \times 100\%$$

$$S_{CO_2} = f_{CH_4-CO_2} \times \frac{area_{CO_2-TCD}}{area_{CH_4-TCD}} \times S_{CH_4} \times 100\%$$

$$S_{C_xH_y} = \frac{area_{C_xH_y-FID}}{\sum area_{C_xH_y-FID}} \times 100\%$$

Supplementary Figures

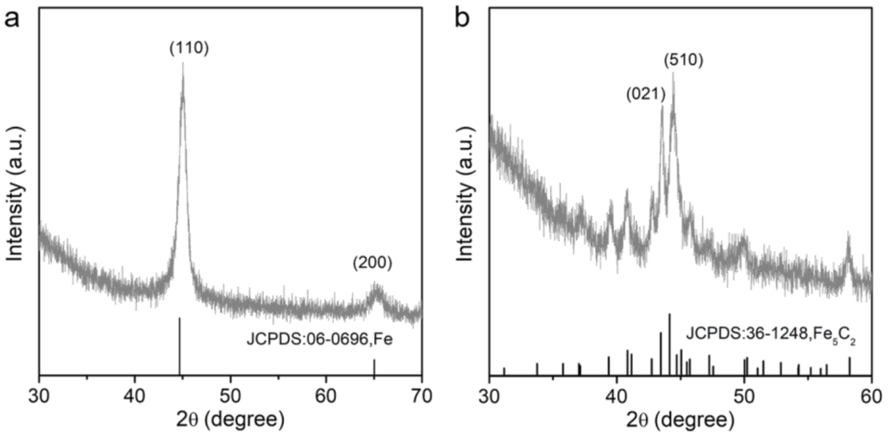

Figure S1. XRD patterns of Fe and pristine Fe_5C_2.

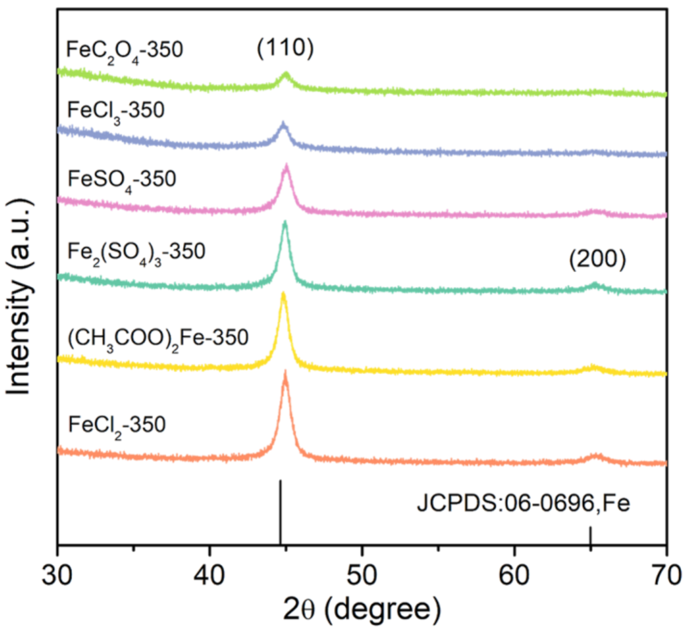

Figure S2. XRD patterns of Fe prepared from different iron salts.

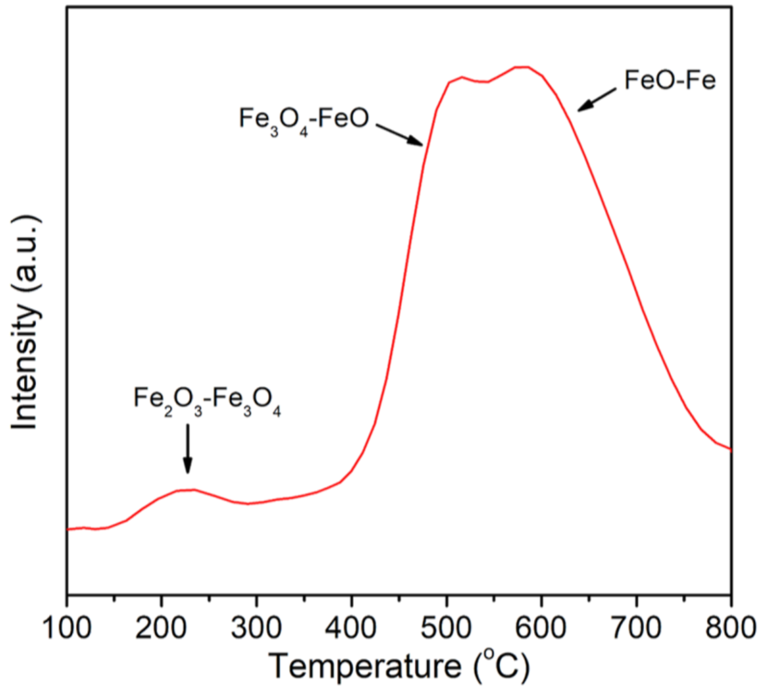

Figure S3. H_2-TPR patterns of NZVI.

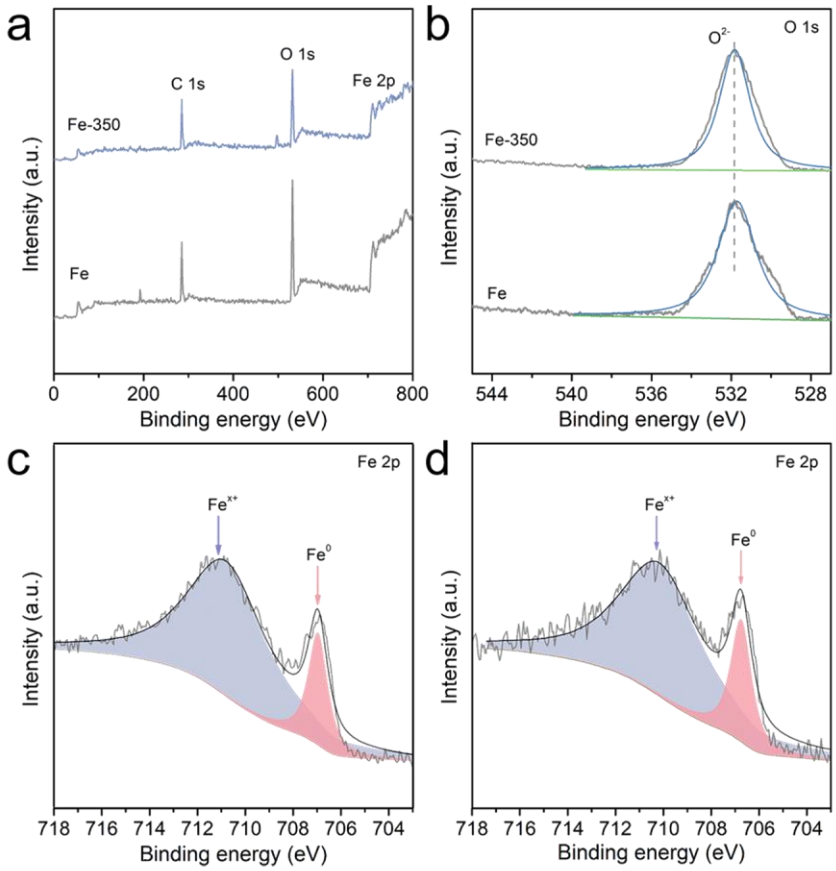

Figure S4. (a) XPS survey spectra and (b) O 1s XPS spectra for Fe and Fe-350 precursors.

Fe 2p XPS spectra for precursors (c) Fe and (d) Fe-350.

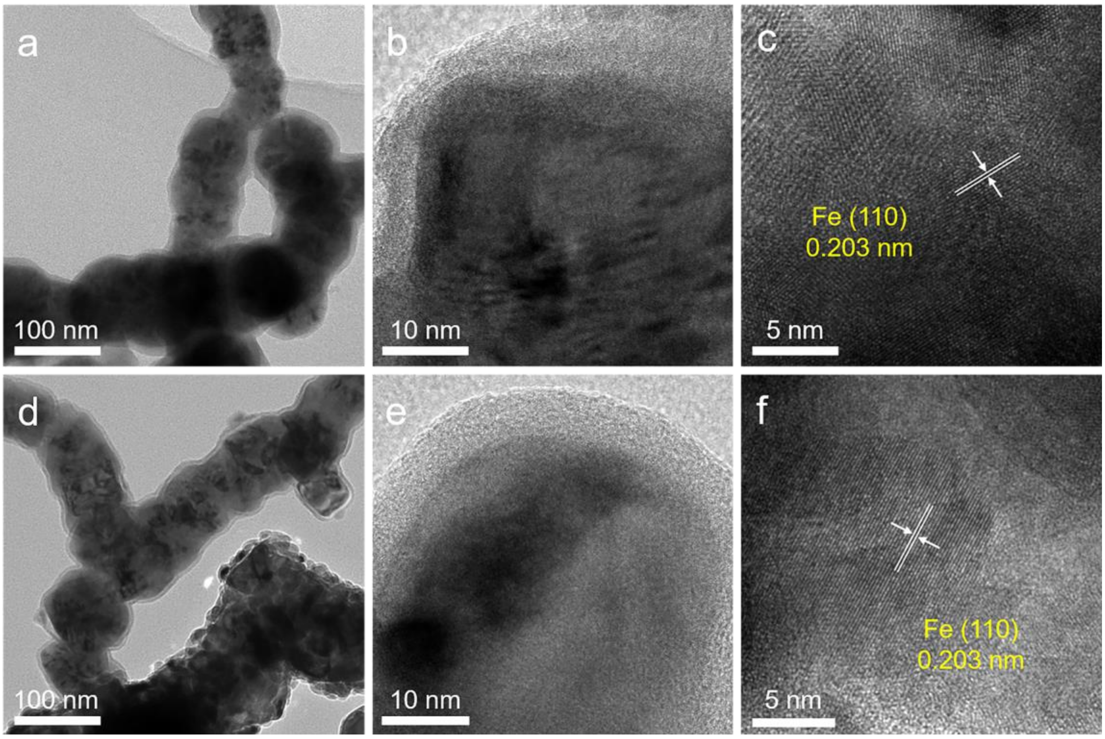

Figure S5. TEM images of Fe-300 and Fe-400. (a) TEM images, (b) and (c) HRTEM images for Fe-300. (d) TEM images, (e) and (f) HRTEM images for Fe-400.

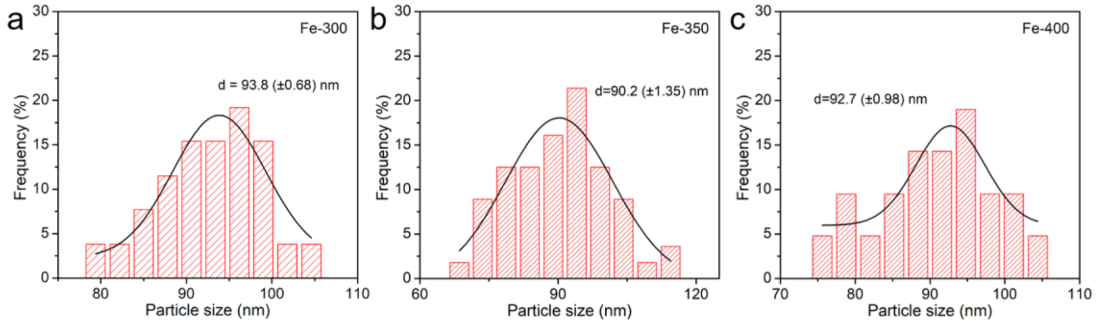

Figure S6. Particle size distribution histograms of Fe-T.

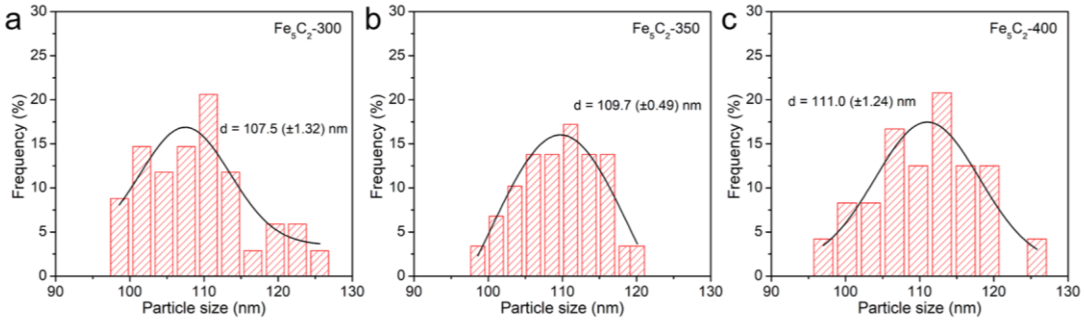

Figure S7. Particle size distribution histograms of Fe_5C_2-T.

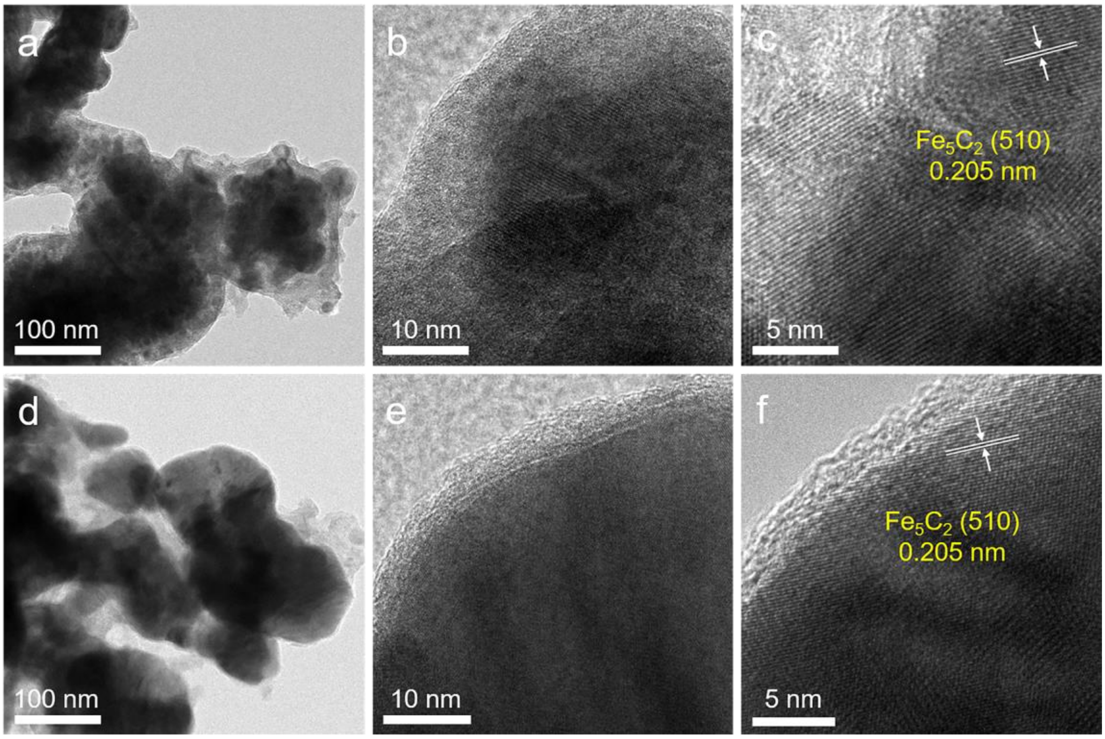

Figure S8. TEM images of Fe_5C_2-300 and Fe_5C_2-400 catalysts. (a) TEM images, (b) and (c) HRTEM images for Fe_5C_2-300 catalyst. (d) TEM images, (e) and (f) HRTEM images for Fe_5C_2-400 catalyst.

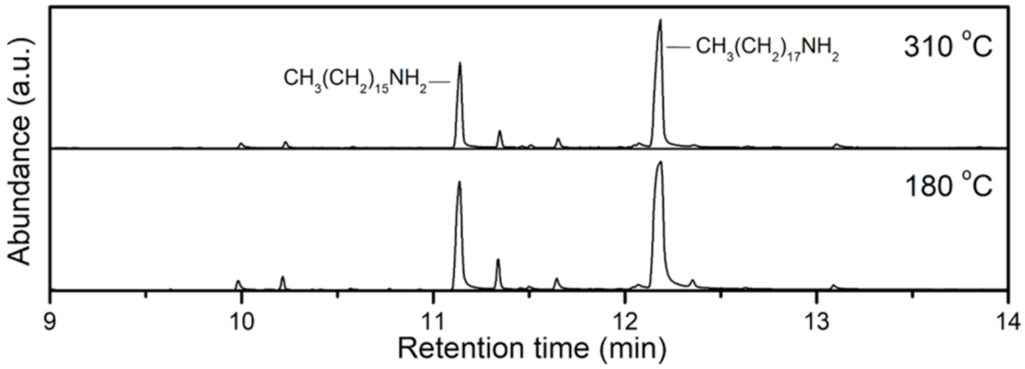

Figure S9. GC-MS chromatograms obtained from the reaction solutions extracted at 180 ^{o}C and 310 ^{o}C.

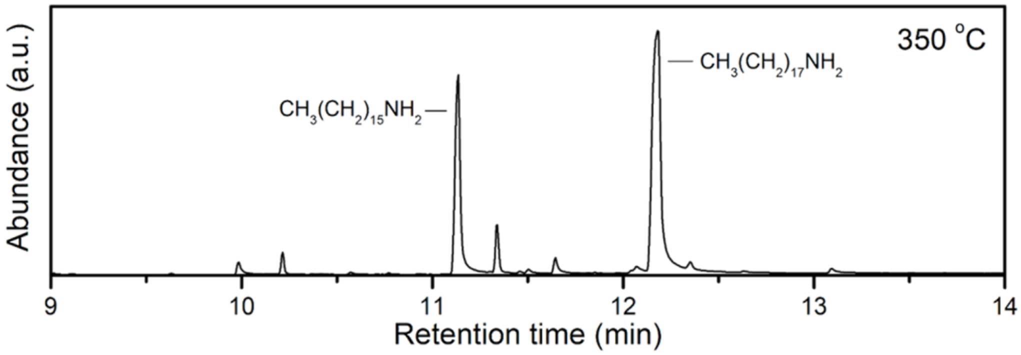

Figure S10. GC-MS chromatograms obtained from the reaction solutions of the blank experiment at 350 °C without NZVI.

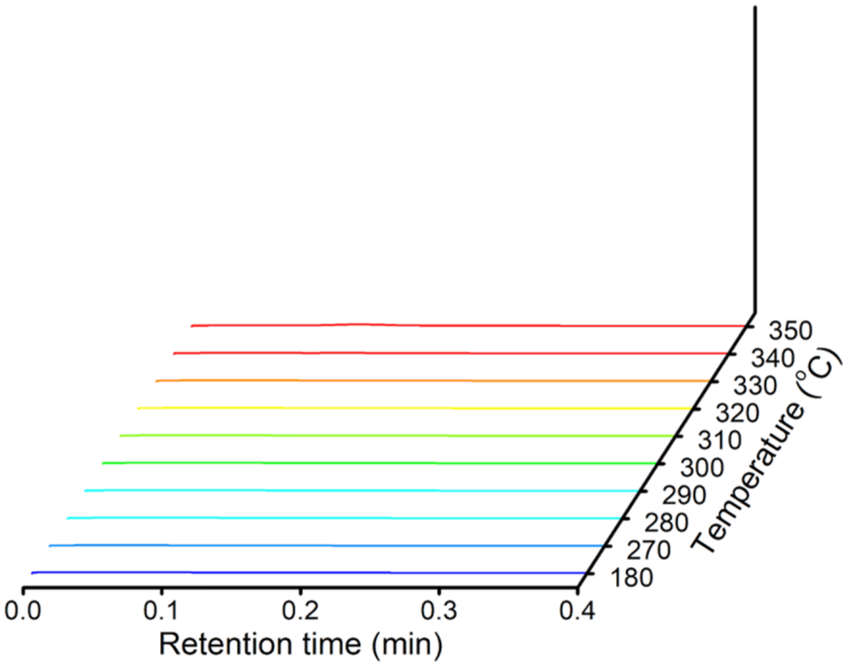

Figure S11. GC chromatograms obtained from the wet synthesis process without NZVI detected by the TCD detector.

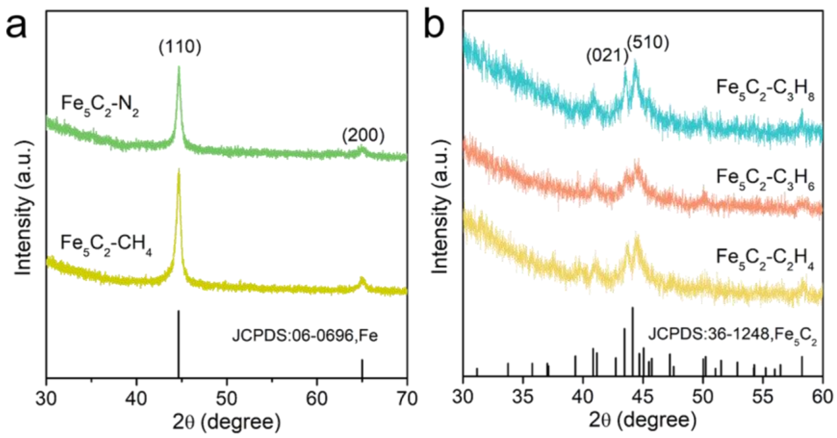

Figure S12. XRD patterns of products generated from control experiments.

Supplementary discussion: The injected gases were methane (CH_4/He = 10/90), ethylene (C_2H_4/He = 10/90), propylene (C_3H_6/N_2 = 10/90) and propane (C_3H_8/N_2 = 10/90), respectively.

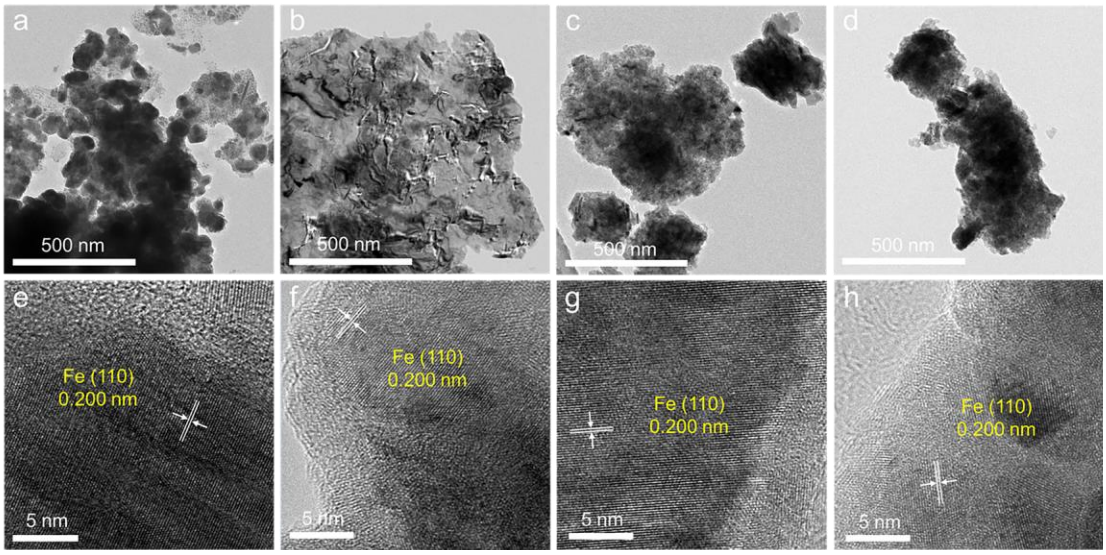

Figure S13. Characterization of Mx-Fe precursors. TEM images of (a) M0-Fe, (b) M10-Fe, (c) M12-Fe and (d) M72-Fe. HRTEM images of (e) M0-Fe, (f) M10-Fe, (g) M12-Fe and (h) M72-Fe.

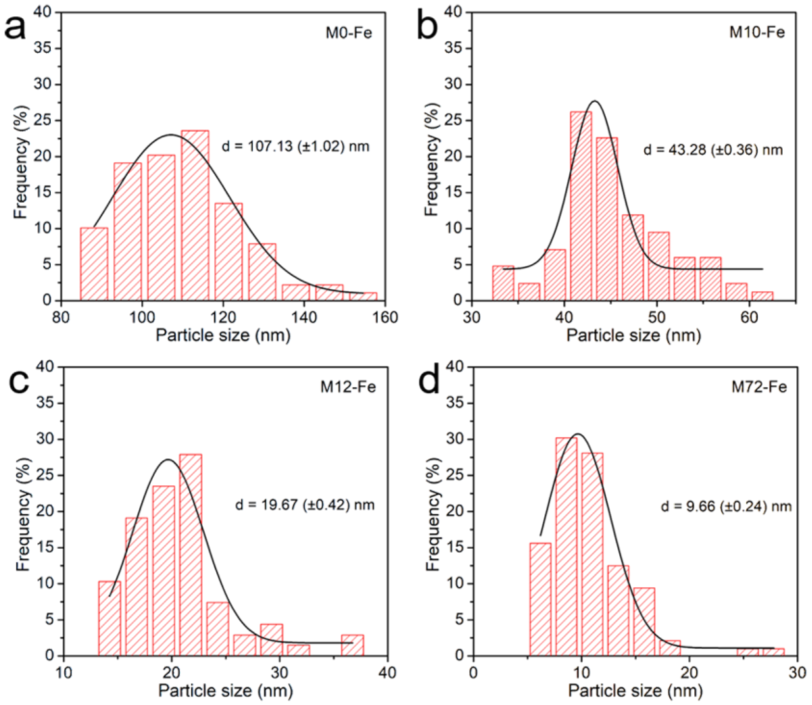

Figure S14. Particle size distribution histograms of Mx-Fe.

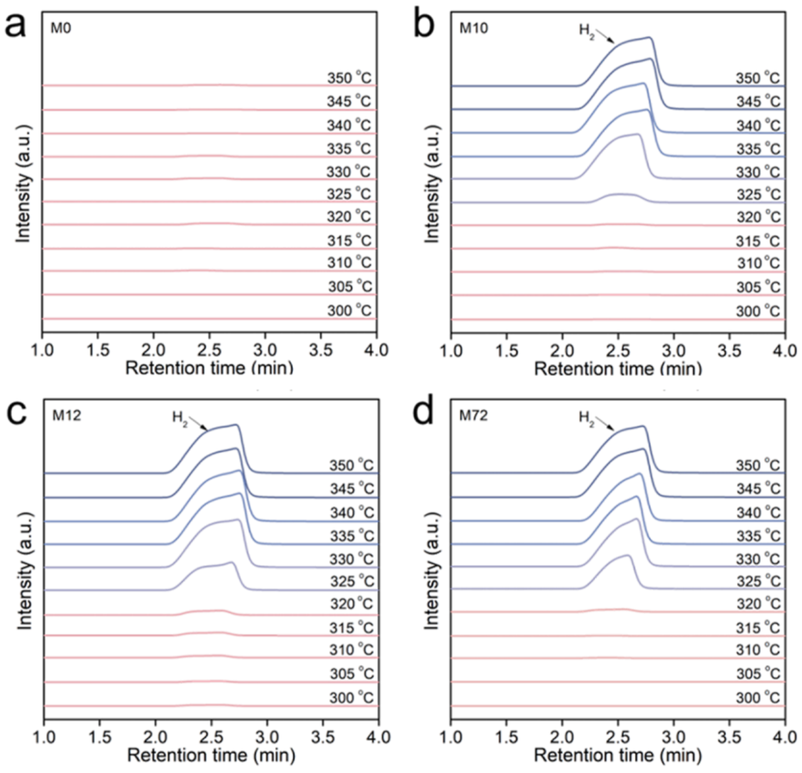

Figure S15. GC chromatograms of the H_2 signal in the wet chemical synthesis process of Mx-Fe.

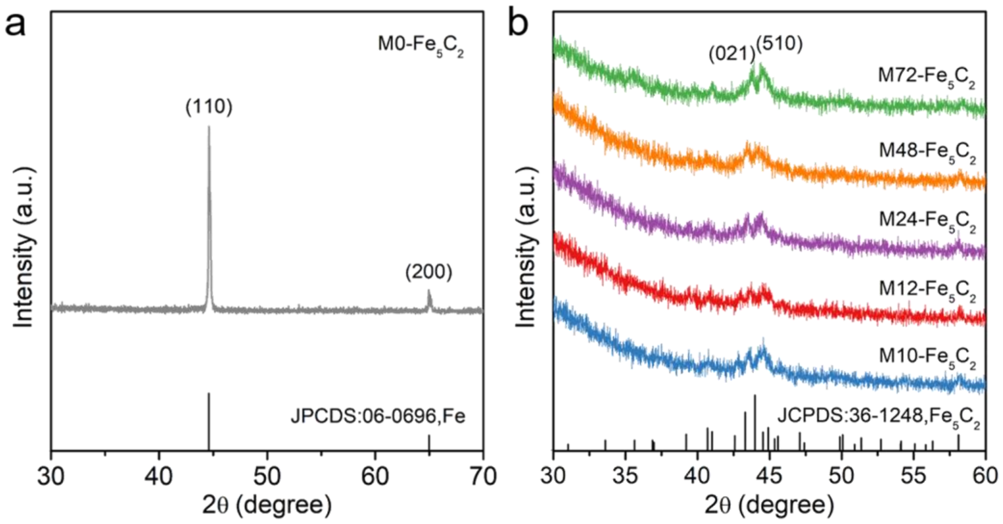

Figure S16. XRD patterns of Mx-Fe_5C_2.

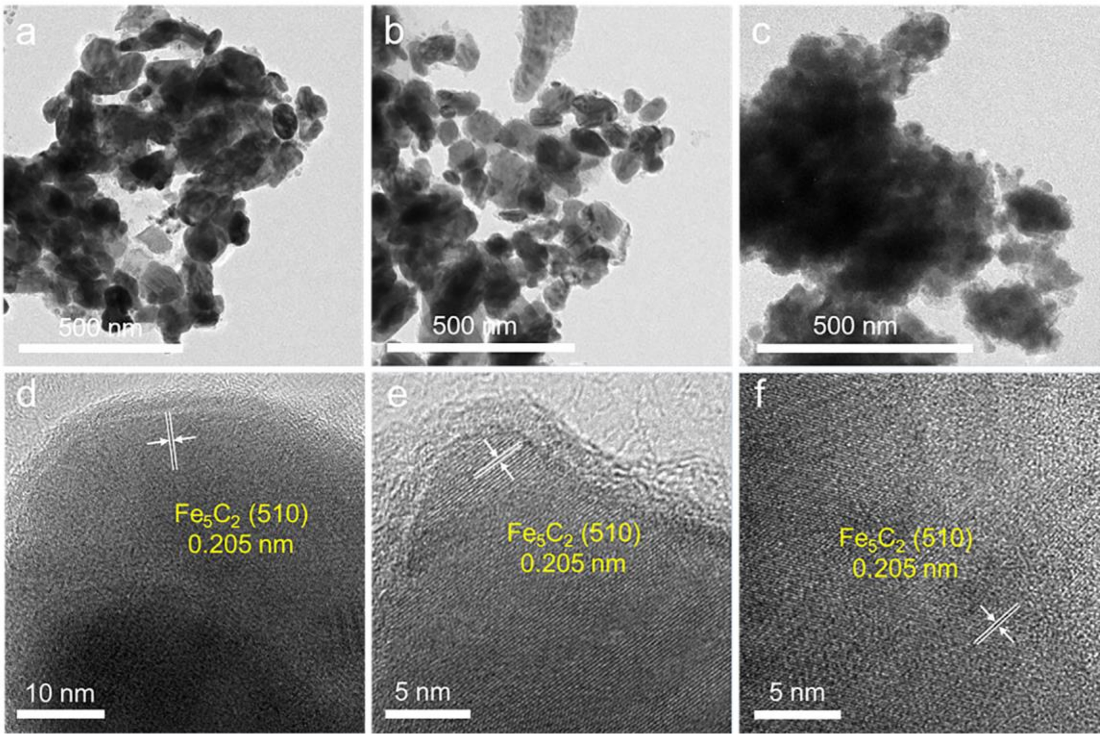

Figure S17. Characterization of Mx-Fe_5C_2 catalysts. TEM images of (a) M10-Fe_5C_2, (b) M12-Fe_5C_2 and (c) M72-Fe_5C_2. HRTEM images of (d) M10-Fe_5C_2, (e) M12-Fe_5C_2 and (f) M72-Fe_5C_2.

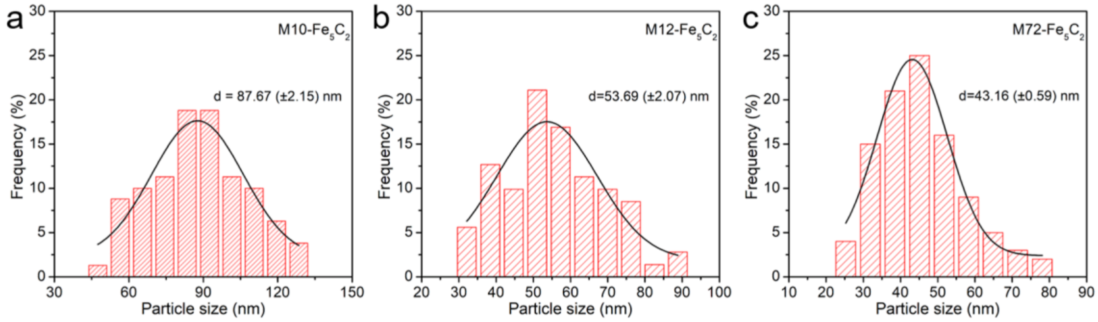

Figure S18. Particle size distribution histograms of Mx-Fe_5C_2.

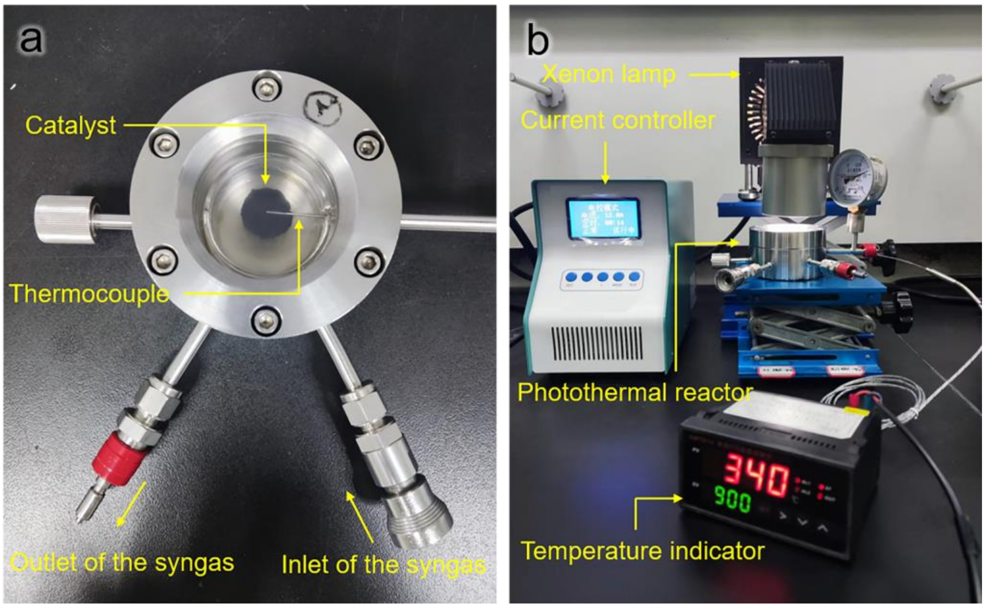

Figure S19. Photographs of (a) photothermal reactor and (b) catalytic evaluation system.

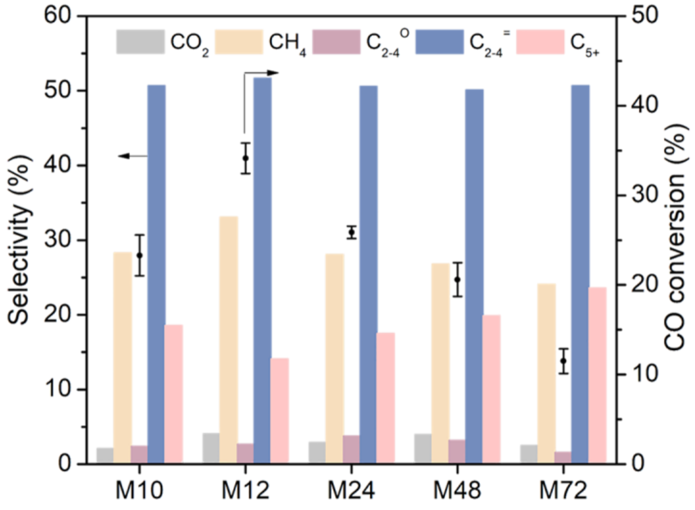

Figure S20. FTS catalytic performance tests of Mx-Fe_5C_2 catalysts.

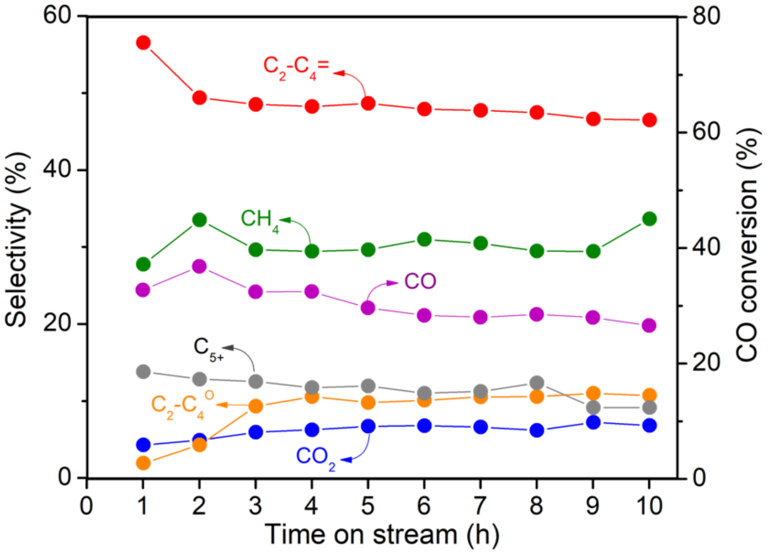

Figure S21. Stability test of M12-Fe_5C_2 catalyst in a flow reaction system for 10 h.

Reaction conditions: catalyst mass, 100 mg; reactant atmosphere, $CO/H_2/N_2$ = 20/60/20, 0.18 MPa; reaction temperature, 340 ^oC.

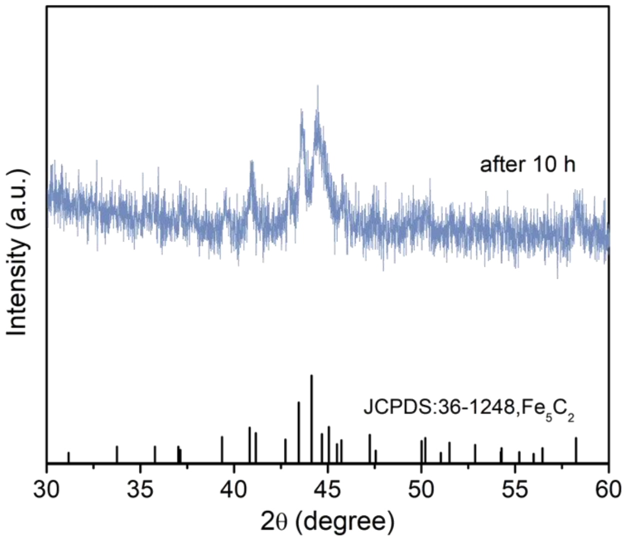

Figure S22. XRD patterns for M12-Fe_5C_2 before the reaction and after 10 h.

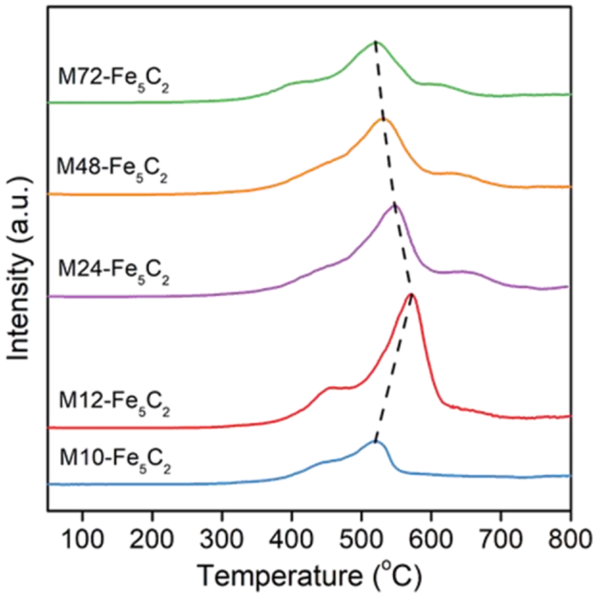

Figure S23. CO temperature-programmed desorption profiles of Mx-Fe_5C_2 catalysts.

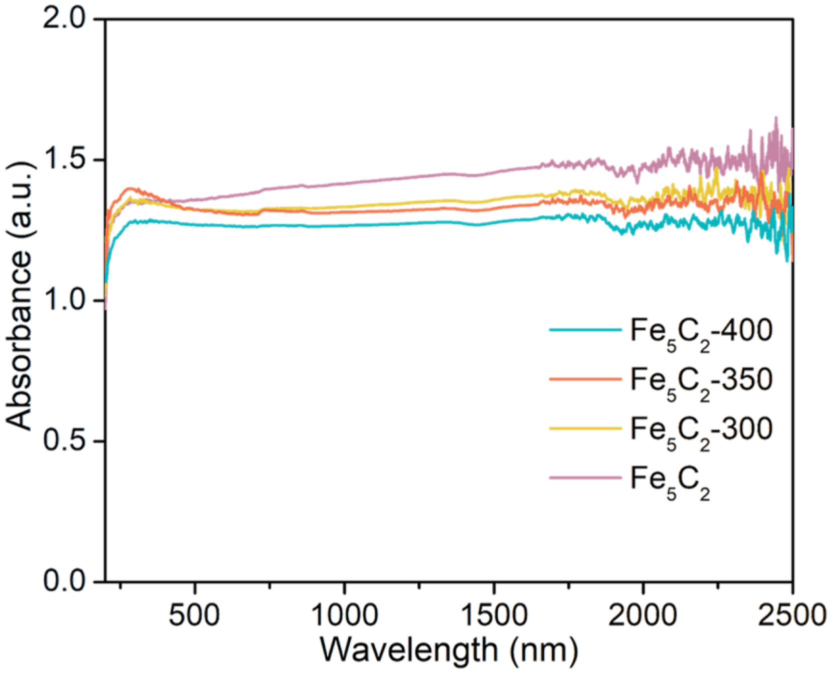

Figure S24. Ultraviolet-visible near-infrared absorption spectra of the Fe_5C_2 and Fe_5C_2-T catalysts.

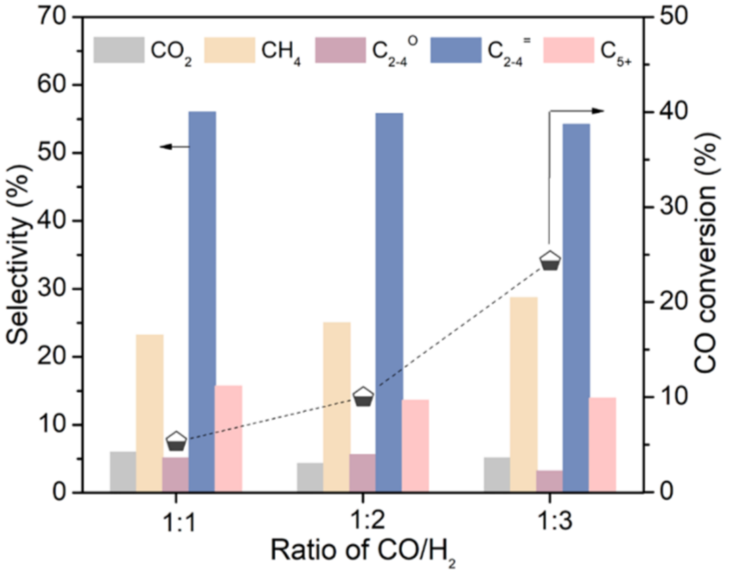

Figure S25. The photothermocatalytic performance of Fe_5C_2-350 catalyst under different ratio of CO/H_2.

Reaction conditions: reactant atmosphere, 0.18 MPa; catalyst mass, 50 mg; irradiation time, 0.5 h; light source, 300 W Xe lamp (λ = 200−1200 nm, 340 °C).

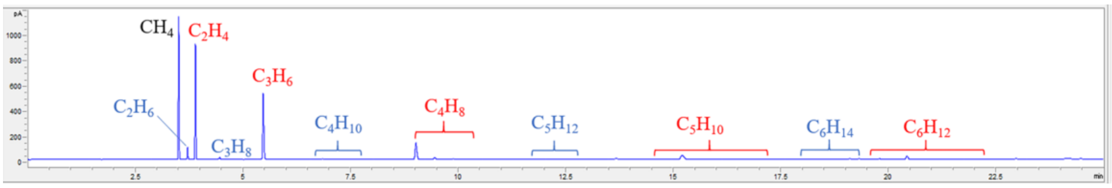

Figure S26. Chromatogram of the hydrocarbon products obtained by CO hydrogenation over the Fe_5C_2-350 catalyst for 0.5 h under light irradiation from a 300 W Xe lamp.

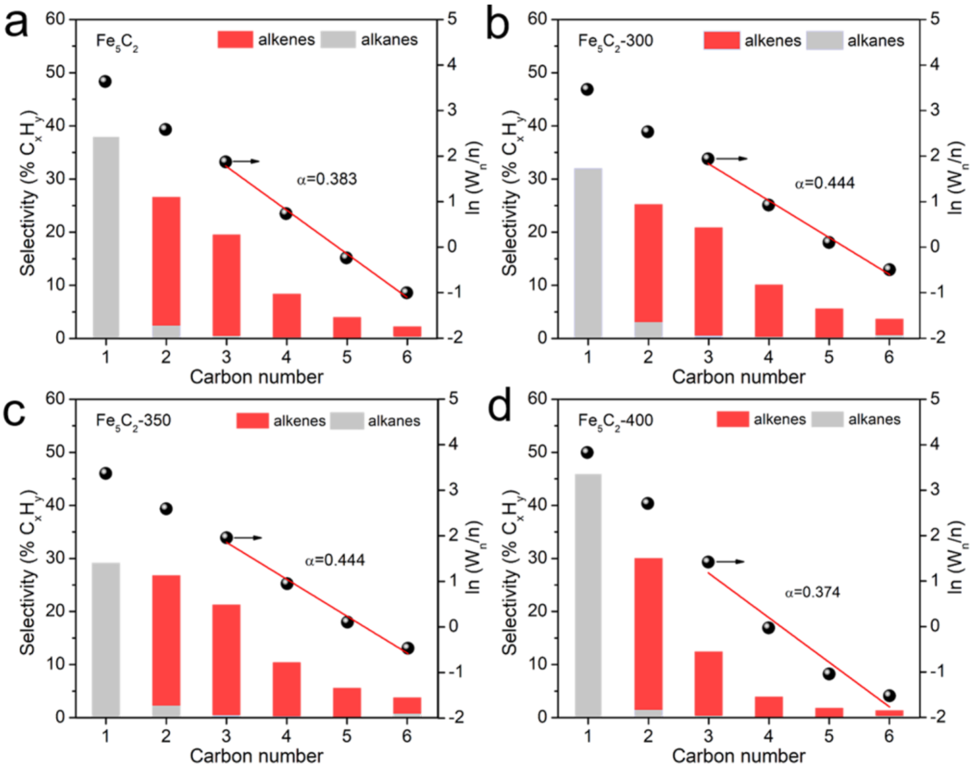

Figure S27. The hydrocarbon product distribution obtained over the catalysts under the irradiation of the 300 W Xe lamp: (a) Fe_5C_2, (b) Fe_5C_2-300, (c) Fe_5C_2-350, (d) Fe_5C_2-400.

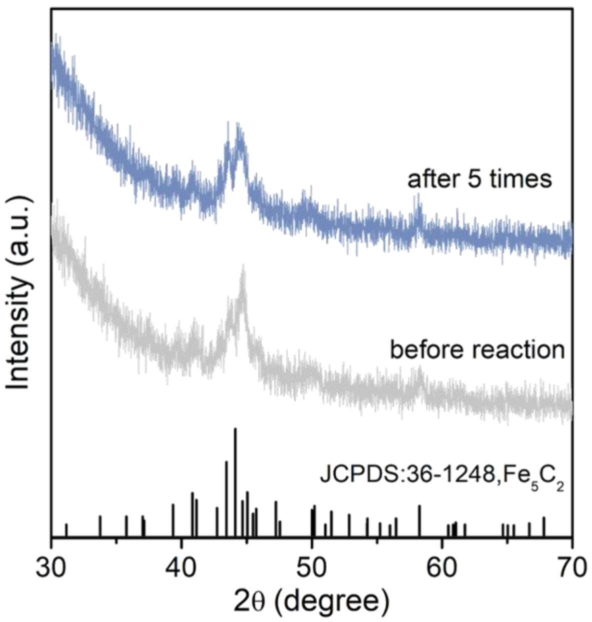

Figure S28. XRD patterns for Fe_5C_2-350 before the reaction and after 5 times.

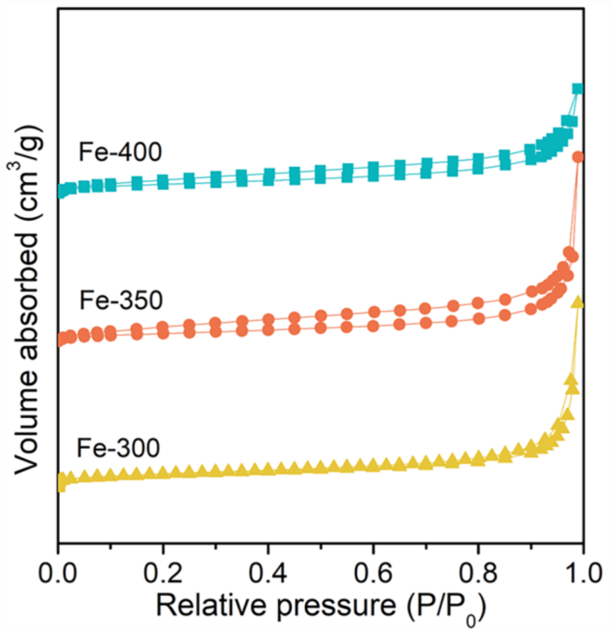

Figure S29. N_2 adsorption-desorption curves of Fe-T samples.

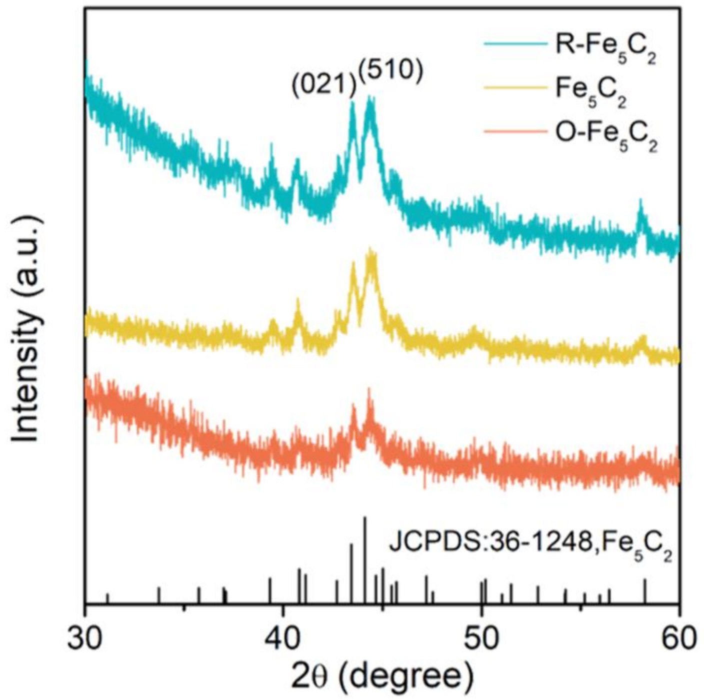

Figure S30. XRD patterns of Fe_5C_2 with different surface oxide states.

Supplementary discussion: The catalyst obtained by the H_2 reduction of Fe_5C_2-350 was named R-Fe_5C_2, and the catalyst obtained by the oxidation of Fe_5C_2-350 in air was denoted as O-Fe_5C_2.

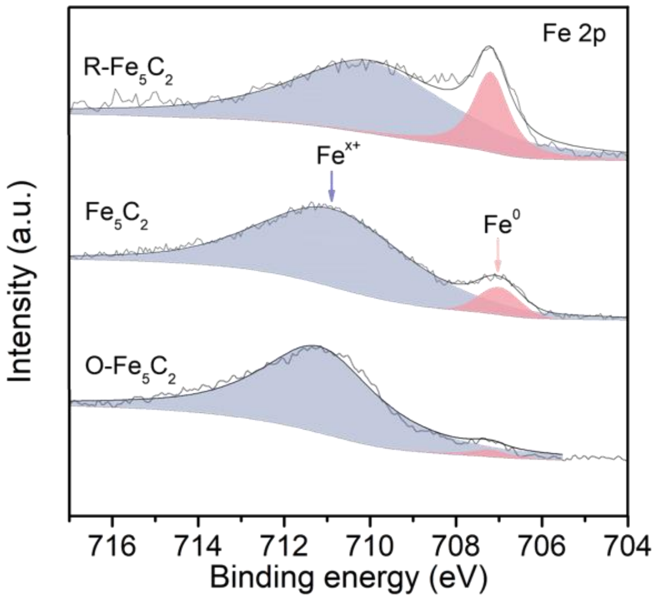

Figure S31. Fe 2p XPS spectra of Fe_5C_2 with different surface oxide states.

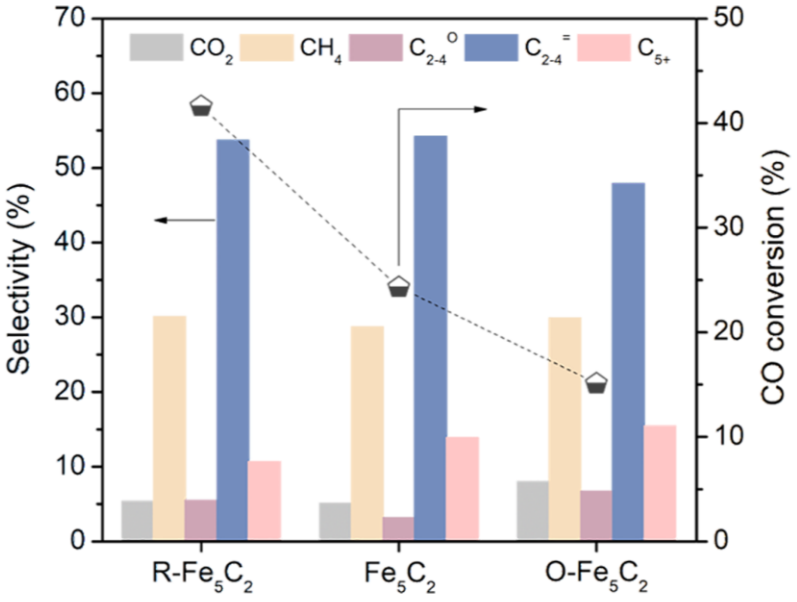

Figure S32. Catalytic performances of Fe_5C_2 catalysts with different surface oxide states.

Reaction conditions: catalyst mass, 50 mg; reactant atmosphere, $CO/H_2/N_2$ = 20/60/20, 0.18 MPa; reaction temperature, 340 ^oC by 300 W Xe lamp irradiation (full solar spectrum), with no external heat input.

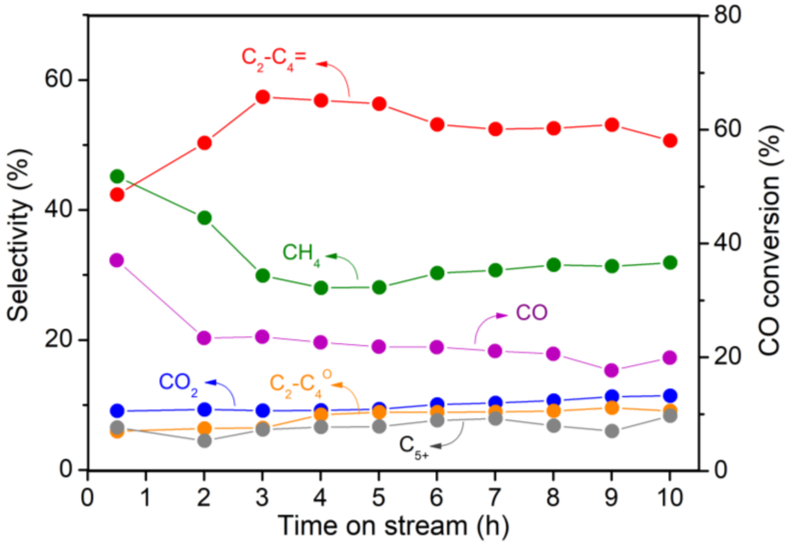

Figure S33. Stability test of Fe_5C_2 prepared from $Fe_2(CO)_9$ in a flow reaction system for 10 h.

Reaction conditions: catalyst mass, 100 mg; reactant atmosphere, $CO/H_2/N_2$ = 20/60/20, 0.18 MPa; reaction temperature, 340 ^oC.

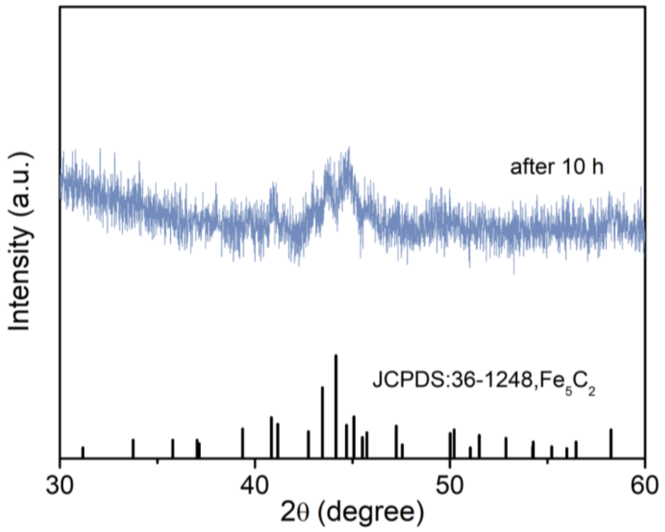

Figure S34. XRD patterns of Fe_5C_2 prepared from $Fe_2(CO)_9$ after the reaction for a duration of 10 h.

Supplementary Tables

Table S1. The price of each material for synthesizing Fe_5C_2.

Materials	CAS	Purity (%)	Weight or volume (g or L)	Price ($\$\cdot g^{-1}$ or $\$\cdot L^{-1}$)
$NaBH_4$	16940-66-2	98.0	500	0.41
$Fe_2(CO)_9$	15321-51-4	98.0	50	10.46
$C_{18}H_{39}N$	124-30-1	90.0	500	0.22
$C_{19}H_{42}BrN$	57-09-0	≥99.0	500	0.36
C_2H_5OH	64-17-5	≥99.5	25	19.32
C_6H_{14}	110-54-3	≥95.0	16	42.63
Fe	7439-89-6	98.0	500	0.08
$FeSO_4\cdot 7H_2O$	7782-63-0	≥99.0	1000	0.11
$FeCl_3\cdot 6H_2O$	10025-77-1	≥99.0	250	0.61
$Fe_2(SO_4)_3\cdot xH_2O$	15244-10-7	97.0	500	0.16
$FeCl_2$	7758-94-3	99.9	50	8.88
$(CH_3COO)_2Fe$	3094-87-9	95.0	50	10.94
$FeC_2O_4\cdot 2H_2O$	6047-25-2	99.0	500	0.12

Supplementary discussion: The above prices were from the Sigma Aldrich company on 8 January 2024, except the price of $NaBH_4$, which was from the Aladdin company.

Table S2. Comparison of the experimental cost between $Fe_2(CO)_9$, iron powder and iron salts.

Cost of the synthesis	60 mg Fe_5C_2 was synthesized with $Fe_2(CO)_9$	150 mg Fe_5C_2 was synthesized with iron powder	150 mg Fe_5C_2 was synthesized with $FeSO_4·7H_2O$
Precursor	0.655 g $Fe_2(CO)_9$: 10.46*0.655 = $6.85	0.2 g Fe: 0.08*0.20 = $0.016	2*1.113 g $FeSO_4·7H_2O$: 0.11*2*1.113 = $0.24<br>2*0.378 g $NaBH_4$: 0.41*2*0.378 = $0.31
Wet chemical process	14.5 g $C_{18}H_{39}N$: 0.22*14.5 = $3.19<br>0.113 g $C_{19}H_{42}BrN$: 0.36*0.113 = $0.04		
Wash and dry catalysts	100 mL C_2H_5OH: 19.32*0.10 = $1.93<br>100 mL C_6H_{14}: 42.93*0.10 = $4.29		
Total cost ($\$·g^{-1}$)	16.30/0.06 = $271.67	9.47/0.15 = $63.12	10.00/0.15 = $66.67

Table S3. Element contents of Fe_5C_2-T catalysts were detected by ICP.

Samples	Content (wt%)		
	Fe	B	Na
Fe_5C_2-300	65.91	1.63	0.24
Fe_5C_2-350	59.66	1.46	0.21
Fe_5C_2-400	63.59	1.39	0.70

Table S4. Element contents of the Fe and Fe-350 samples.

Samples	XPS atom %		
	Fe	O	C
Fe	7.8	46.0	46.2
Fe-350	7.2	36.4	56.4

Supplementary discussion: The XPS results demonstrated that the ratio of the peak area of Fe^{x+} to Fe^{0} of sample Fe was 3.0, while that of sample Fe-350 was 2.3.

Table S5. Element contents of the M12-Fe and M72-Fe samples.

Samples	XPS atom %		
	Fe	O	C
M12-Fe	9.0	39.5	51.5
M72-Fe	11.3	39.1	49.6

Table S6. Catalytic performance of blank experiment and Fe-350 sample.

Entry[a]	Samples	CO conv. (%)	CO_2 sel. (%)	Hydrocarbon sel. (%, CO_2 free)			
				CH_4	$C_{2-4}^{=}$	C_{2-4}^{0}	C_5^{+}
1	No-catalyst	0	-	-	-	-	-
2	Fe-350	5.1	19.0	38.7	15.3	35.3	10.7

[a]Reaction conditions: reaction atmosphere, $CO/H_2/N_2$ = 20/60/20, 0.18 MPa; catalyst mass, 50 mg; irradiation time, 0.5 h; light source, 300 W Xe lamp (λ = 200−1200 nm, 340 °C).

Table S7. Surface area and pore volume dates for Fe-T samples.

Samples	BET surface area ($m^2 \cdot g^{-1}$)	Pore volume ($cm^3 \cdot g^{-1}$)
Fe-300	8.100	0.048
Fe-350	7.548	0.048
Fe-400	5.933	0.026

Table S8. Catalytic performance of Fe_5C_2 obtained from $Fe_2(CO)_9$ or iron powder.

Iron source	CO conv. (%)	CO_2 sel. (%)	Hydrocarbon sel. (%, CO_2 free)			
			CH_4	$C_{2-4}^{=}$	C_{2-4}^{0}	C_5^{+}
$Fe_2(CO)_9$	35.8	8.0	31.9	50.2	5.7	12.2
Iron powder	34.1	4.1	33.1	51.7	2.7	14.1

Reaction conditions: reaction atmosphere, $CO/H_2/N_2$ = 20/60/20, 0.18 MPa; catalyst mass, 50 mg; irradiation time, 0.5 h; light source, 300 W Xe lamp (λ = 200−1200 nm, 340 °C).

Reference

[1] H. Chen, S. Ouyang, M. Zhao, Y. Li, J. Ye, *ACS Appl. Mater. Interfaces* **2017**, *9*, 40333-40343.

[2] Y. Li, R. Li, Z. Li, W. Wei, S. Ouyang, H. Yuan, T. Zhang, *Chem. Res. Chin. Univ.* **2020**, *36*, 1006-1012.

[3] R. Li, Y. Li, Z. Li, W. Wei, Q. Hao, Y. Shi, S. Ouyang, H. Yuan, T. Zhang, *ACS Catal.* **2022**, *12*, 5316-5326.
